# Characterization of two flavonol synthases with iron-independent flavanone 3-hydroxylase activity from *Ornithogalum caudatum* Jacq

**DOI:** 10.1186/s12870-019-1787-x

**Published:** 2019-05-14

**Authors:** Yu-Jia Sun, Jiu-Ming He, Jian-Qiang Kong

**Affiliations:** Institute of Materia Medica, Chinese Academy of Medical Sciences & Peking Union Medical College (State Key Laboratory of Bioactive Substance and Function of Natural Medicines & NHC Key Laboratory of Biosynthesis of Natural Products), Beijing, 100050 China

**Keywords:** Flavonol synthase, *Ornithogalum caudatum*, Flavanone 3-hydroxylase

## Abstract

**Background:**

Flavonol synthase (FLS) is the key enzyme responsible for the biosynthesis of flavonols, the most abundant flavonoids, which have diverse pharmaceutical effects. Flavonol synthase has been previously found in other species, but not yet in *Ornithogalum caudatum*.

**Results:**

The transcriptome-wide mining and functional characterisation of a flavonol synthase gene family from *O. caudatum* were reported. Specifically, a small FLS gene family harbouring two members, OcFLS1 and OcFLS2, was isolated from *O. caudatum* based on transcriptome-wide mining. Phylogenetic analysis suggested that the two proteins showed the closest relationship with FLS proteins. In vitro enzymatic assays indicated OcFLS1 and OcFLS2 were flavonol synthases, catalysing the conversion of dihydroflavonols to flavonols in an iron-dependent fashion. In addition, the two proteins were found to display flavanone 3β-hydroxylase (F3H) activity, hydroxylating flavanones to form dihydroflavonols. Unlike single F3H enzymes, the F3H activity of OcFLS1 and OcFLS2 did not absolutely require iron. However, the presence of sufficient Fe^2+^ was demonstrated to be conducive to successive catalysis of flavanones to flavonols. The qRT-PCR analysis demonstrated that both genes were expressed in the leaves, bulbs, and flowers, with particularly high expression in the leaves. Moreover, their expression was regulated by developmental and environmental conditions.

**Conclusions:**

OcFLS1 and OcFLS2 from *O. caudatum* were demonstrated to be flavonol synthases with iron-independent flavanone 3-hydroxylase activity.

**Electronic supplementary material:**

The online version of this article (10.1186/s12870-019-1787-x) contains supplementary material, which is available to authorized users.

## Background

**Fig. 1:**
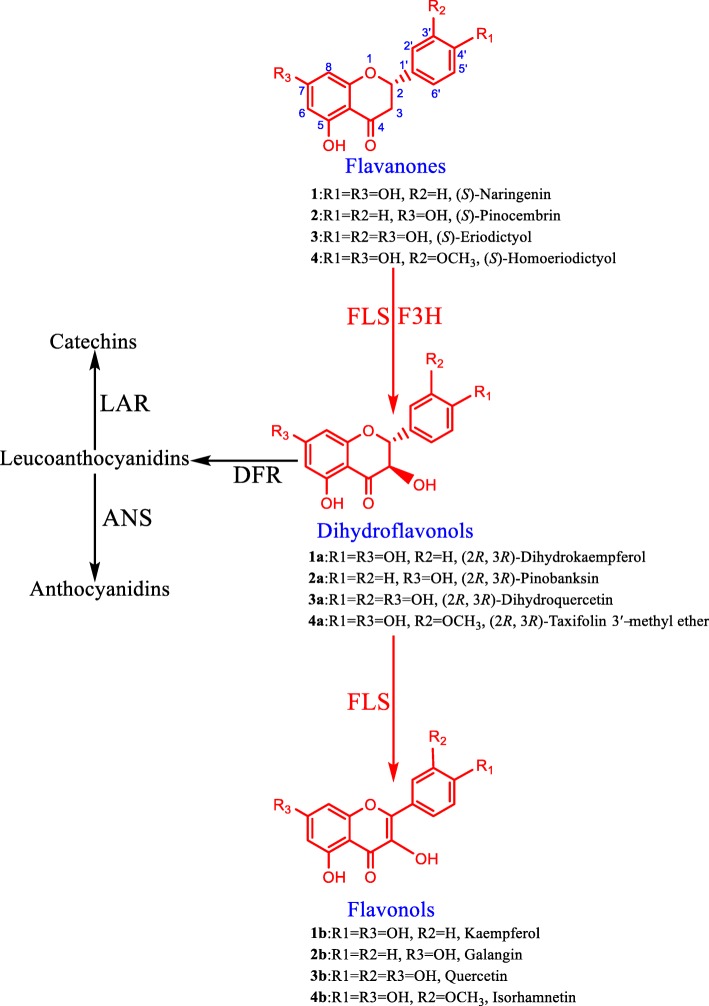
Biosynthetic pathways catalysed by flavonol synthases. *F3H* flavanone 3-hydroxylase, *FLS* flavonol synthase, *DFR* dihydroflavonol 4-reductase, *ANS* anthocyanidin synthase, *LAR* leucoanthocyanidin reductase

Flavonols are the most abundant and widely distributed flavonoids in nature [1]. Flavonols display a wide range of biological activity, such as antioxidative effects [2–6], anti-cancer activities [7–9], anti-inflammatory properties [10–12], and antidiabetic actions [13–15]. The diverse activities make flavonols promising molecules for drug development and the biosynthesis of flavonols is thus attracting increasing attention from researchers [16–19]. The biosynthesis of flavonols starting from flavanones includes two steps (Fig. 1). First, flavanones are hydroxylated by flavanone 3-hydroxylase (F3H) to yield dihydroflavonols, which might be converted subsequently to flavonols by the introduction of a double bond between C-2 and C-3 through the action of a flavonol synthase (FLS), a non-heme ferrous enzyme that belongs to a family of 2-oxoglutarate-dependent dioxygenase (2-ODD). Alternatively, dihydroflavonols might be reduced to leucoanthocyanidins by dihydroflavonol 4-reductase (DFR) en route to the formation of catechins and anthocyanidins (Fig. 1). Thus, FLSs are located at the bifurcation branches, diverting metabolic flux to flavonols. Besides FLS activity, flavonol synthases exhibit F3H activity, accepting flavanones as substrates [18]. FLSs are therefore regarded as flavonoid 2-ODDs with broad substrate specificity and represent the key structural proteins in the biosynthesis of flavonols (Fig. 1).

FLS was first identified in parsley suspension cultures by Britsch et al. [20] as a dioxygenase enzyme. Subsequently, FLS was demonstrated to be widespread in various species, such as *Petroselinum hortense* Hoffm. [20], *Litchi chinensis* [21], *Cyclamen purpurascens* [22], among others. Because of their vital role in flavonol biosynthesis, FLS genes were introduced into varied microbes to construct engineered cells for green preparation of flavonols [23–31]. However, the actual output of target flavonols in these engineered strains is insufficient for industrial production. One strategy to increase the flavonol yield in these cell factories lies in the application of flavonol synthases with higher catalytic activity. Thus, isolation of new FLS genes from different organisms, such as *Ornithogalum caudatum*, is an effective way to identify superior candidates with better catalytic activity.

*Ornithogalum caudatum* is a medicinal plant rich in flavonols and their glycosides, such as quercetin, kaempferol, isorhamnetin, quercetin 3-*O*-β-D-glucopyranoside, kaempferol 3-*O*-β-D-glucopyranoside, rutin, and kaempferol 3-*O*-β-rutinoside [32], suggesting that this species contains genes encoding flavonol synthase. Therefore, this plant was chosen as the starting material for FLS gene isolation. Herein, a transcriptome-wide mining and functional characterisation of a flavonol synthase gene family from *O. caudatum* are reported. Specifically, a small FLS gene family containing two members was isolated from *O. caudatum*. An in vitro enzymatic assay indicated the two FLS enzymes were able to convert dihydroflavonols to flavonols in an iron-dependent fashion. In addition, the two enzymes exerted F3H action, catalysing flavanones to form dihydroflavonols without the absolute requirement of ferrous iron. However, the presence of sufficient Fe^2+^ lead to efficient biotransformation of flavonols from flavanones. The expression profiles of the two genes in the plant revealed that their expression was regulated by developmental and environmental conditions. These findings deepen our understanding of flavonol synthase, thereby broadening its application.

## Results

### In silico identification of unigenes coding for FLS

After functional annotation, four unigenes, namely unigenes 61,710, 27,929, 76,508, and 101,341, showing high sequence similarity with flavonol synthase were retrieved from RNA-seq data (Additional file 1: Figure S1). Unigene 61,710 was 1280 bp long and contained an ORF of 1008 bp (Additional file 1: Figure S1). In addition, this unigene contained a 5′-untranslated region (5′-UTR) of 81 bp and a 3′-UTR of 191 bp (Additional file 1: Figure S1). The other three unigenes, namely unigene 27,929, 76,508, and 101,341, were 878, 246, and 298 bp, respectively (Additional file 1: Figure S1). None of the unigenes contained a full-length ORF. Sequence alignments revealed that unigene 27,929, 76,508, and 101,341 displayed sequence identities with 5′-end, 3′-end and middle portion of the *FLS* gene, respectively (Additional file 1: Figure S1). Unigene 27,929 contained a 5′-UTR of 52 bp and a 5′-region of ORF with 826 bp. Unigene 76,508 had a region of 188 bp, 3′-end of an ORF, and a 3′-UTR of 66 bp. Unigene 101,341 was 298 bp long and spanned nucleotide 613 to 910 of the *FLS* gene. Moreover, unigenes 27,929 and 101,341, as well as unigenes 76,508 and 101,341, were observed to have sequence overlaps, suggesting the three unigenes might be located in the same sequence (Additional file 1: Figure S1). These unigenes were thus assembled into a longer unigene with 1126 bp, including a 5′-UTR of 52 bp, an ORF of 1008 bp, and a 3′-UTR of 66 bp (Additional file 1: Figure S1). The data indicated there was a small gene family containing at least two members in the *O. caudatum* genome, one member corresponded to unigene 61,710, whereas the other *FLS* gene consisted of unigenes 27,929, 76,508, and 101,341. Therefore, these two FLS-like unigenes were selected for further investigation.

### cDNA sequence isolation and analyses

To determine the identity of these unigenes, nested PCRs were performed to isolate the corresponding cDNAs using *O. caudatum* cDNA as the template. Using unigene 61,710-specific primers (Additional file 12: Table S1), this PCR-based approach resulted in the amplification of a 1008 bp cDNA fragment designated as OcFLS1 (Additional file 2: Figure S2). Moreover, another PCR fragment termed OcFLS2 was isolated from *O. caudatum* cDNA by a combinational use of a forward primer corresponding to unigene 27,929 and reverse primers specific to unigene 76,508 (Additional file 2: Figure S2). OcFLS1 and OcFLS2 were then cloned into pEASY-Blunt, thereby forming two recombinant plasmids pEASY-OcFLS1 and pEASY-OcFLS2 for sequencing (Additional file 12: Table S2). DNA sequence alignment with software Blast N showed OcFLS1 had 99% identity to unigene 61,710. Additionally, as we had expected, OcFLS2 consisted of unigene 27,929, 76,508, and 101,341 (Additional file 1: Figure S1). This evidence indicated that OcFLS1 and OcFLS2 were bona fide genes in the *O. caudatum* genome. The two sequences were thus submitted to GenBank with accession numbers MH748569 (OcFLS1) and MH748570 (OcFLS2).

OcFLS1 contained an ORF of 1008 bp encoding a protein with 335 amino acids (aa) with a predicted molecular weight of 37.835 kDa and isoelectric point (pI) of 5.57. The full-length ORF of OcFLS2 was also 1008 bp in length. The molecular mass and theoretical pI of OcFLS2 were calculated using the Compute pI/Mw tool (https://web.expasy.org/compute_pi/) as 37.643 kDa and 5.38, respectively. The amino acid sequence encoded by OcFLS1 showed 94% identity to that of OcFLS2.

The TMHMM server predicted that no transmembrane helices were found in OcFLS1 or OcFLS2 (http://www.cbs.dtu.dk/services/TMHMM/#opennewwindow). The two OcFLS proteins showed highly similarity to previously reported FLSs from other plants, such as CpurFLS1(BBA27023.1) and CpurFLS2(BBA024.1) from *Cyclamen purpurascens* [22], AcFLS (AFA55179.1) from *Acacia confuse* [33], FtFLS1(AEC33116.1) from *Fagopyrum tataricum* [34], as well as AtFLS1(NP_196481.1) and AtFLS3(NP_201164.1) from *Arabidopsis thaliana* [35]. The multiple sequence alignment indicated that both of the two proteins possessed the highly conserved ferrous iron binding motif HXDX_53_H (in these motifs X represents any amino acid) and the 2-oxoglutarate binding motif RXS, indicating that both belonged to 2-ODDs (Additional file 3: Figure S3).

A total of 36 flavonoid 2-ODDs were used for phylogenetic analysis (Additional file 4: Figure S4). These 2-ODD sequences were clustered into four distinct clades, namely FSI, F3H, ANS, and FLS. The members in the FSI, F3H, ANS, and FLS clades were sequences coding for flavone synthase I (FSI), flavanone 3-hydroxylase, anthocyanidin synthase (ANS), and flavonol synthase. OcFLS1 and OcFLS2 were clustered in the FLS-related clade with other FLSs. Both showed the closest relationship with FLS proteins from *Allium cepa* (Additional file 4: Figure S4), which correlates with their closer botanical relationship within the monocot taxa.

### Heterologous expression and protein purification

**Fig. 2:**
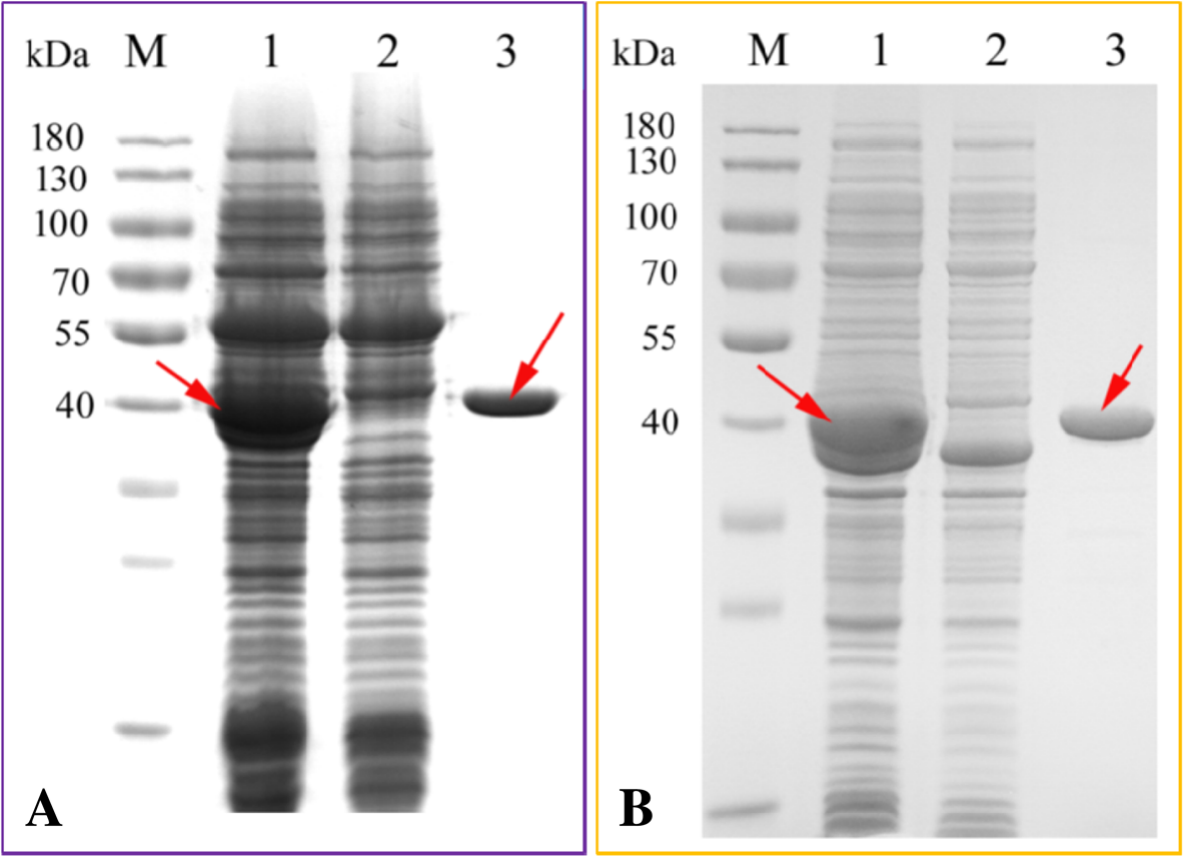
SDS-PAGE analyses of the recombinant OcFLS1 (**a**) and OcFLS2 (**b**). Lane 1, the crude extract expressing the recombinant OcFLS1 (**a**) and OcFLS2 (**b**). Lane 2, the crude extract expressing no recombinant OcFLS1 (**a**) and OcFLS2 (**b**); Lane 3, the purified recombinant OcFLS1 (**a**) and OcFLS2 (**b**);Lane M, the migration of standard protein markers is shown with kDa on the left-hand side of the gel

OcFLS1 and OcFLS2 were subcloned into pET-28a (+) to yield two recombinant constructs pET28a-OcFLS1 and pET28a-OcFLS2 (Additional file 12: Table S2), which were then transformed into *E. coli Trans*etta (DE3) for heterologous expression. The soluble expressions of OcFLS1 and OcFLS2 in *Trans*etta (DE3) were verified using SDS-PAGE. As illustrated in Fig. 2, an intense band representing OcFLS1 was present in the total extract of *Trans*etta (DE3)[pET28a-OcFLS1]. Conversely, no corresponding band was detected in the same position of the control strain *Trans*etta (DE3) [pET-28a (+)], suggesting OcFLS1 was successfully expressed in *E. coli* in a soluble form. Likewise, the soluble expression of OcFLS2 was observable in SDS-PAGE analysis (Fig. 2). The two recombinant proteins were then purified to near homogeneity and the resulting purified proteins were quantified as 32.729 (OcFLS1) and 80.25 mg/ml (OcFLS2) (Fig. 2).

### Flavonol synthase activity of OcFLSs

**Fig. 3:**
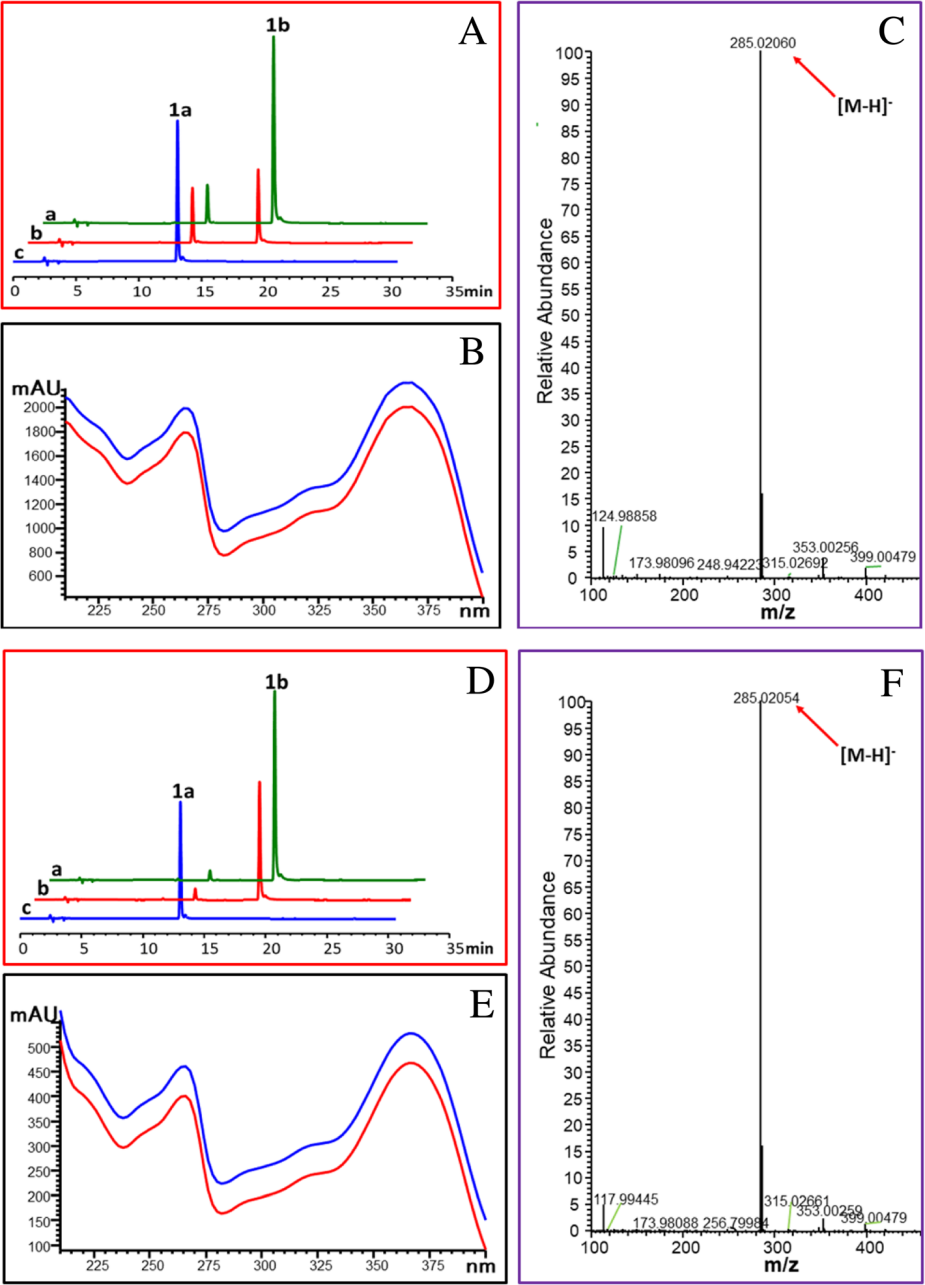
The bioconversion of kaempferol (**1b**) from dihydrokaempferol (**1a**) catalysed by OcFLS1 (**a**-**c**) or OcFLS2 (**d**-**f**). **a**, **d**: HPLC chromatogram of the reaction product of dihydrokaempferol (**1a**) with OcFLS1 (**a**) and OcFLS2 (**d**). **a**, co-elution of the reaction product with authentic kaempferol (**1b**). **b**, the reaction product of dihydrokaempferol (**1a**) with purified protein. **c**, the reaction product of dihydrokaempferol (**1a**) without purified protein. **b**, **e**: UV spectrum of reaction product **1b** (blue line) and authentic kaempferol (red line). **c**, **f**: MS spectrum of reaction product **1b** produced by OcFLS1 (**c**) and OcFLS2 (**f**)

Although OcFLS1 and OcFLS2 were grouped in the flavonol synthase family, comprehensive biochemical characterisation is required because of the high sequence identity between flavonoid-related dioxygenases [36, 37]. Without a special explanation, the biocatalyst used in this study was purified enzymes. To test the FLS activity of OcFLS1, dihydrokaempferol (**1a**) was used as the substrate for the purified OcFLS1 in the presence of α-ketoglutaric acid, ferrous sulphate, and ascorbic acid. After incubation at 20 °C for 2 h, a new peak with a retention time (Rt) of 18.4 min was present in the reaction mixture (Fig. 3a). This peak displayed the same UV spectrum as that of the authentic standard kaempferol (**1b**), suggesting this new product had a similar structure to that of kaempferol (**1b**) (Fig. 3b). Further MS spectrum ([M-H]^−^ ion at *m/z* 285.02060) (Fig. 3c) and co-elution with standard kaempferol (**1b**) (Fig. 3a) confirmed that the new metabolite was kaempferol (**1b**).

Besides dihydrokaempferol (**1a**), other dihydroflavonols, like pinobanksin (**2a**) (Additional file 5: Figure S5), dihydroquercetin (**3a**) (Additional file 6: Figure S6), and 3′-*O*-methyltaxifolin (**4a**) (Additional file 7: Figure S7), could be oxidised by OcFLS1, thereby generating corresponding flavonols [galangin (**2b**), quercetin (**3b**), and isorhamnetin (**4b**)]. However, OcFLS1 had no activity towards other compounds, **5**–**15,** listed in Additional file 8: Figure S8. Similar observations were made for OcFLS2 (Fig. 3 and Additional file 5: Figure S5 Additional file 6: Figure S6 Additional file 7: Figure S7). These data collectively indicated that both enzymes were flavonol synthases, specific for dihydroflavonols.

**Fig. 4:**
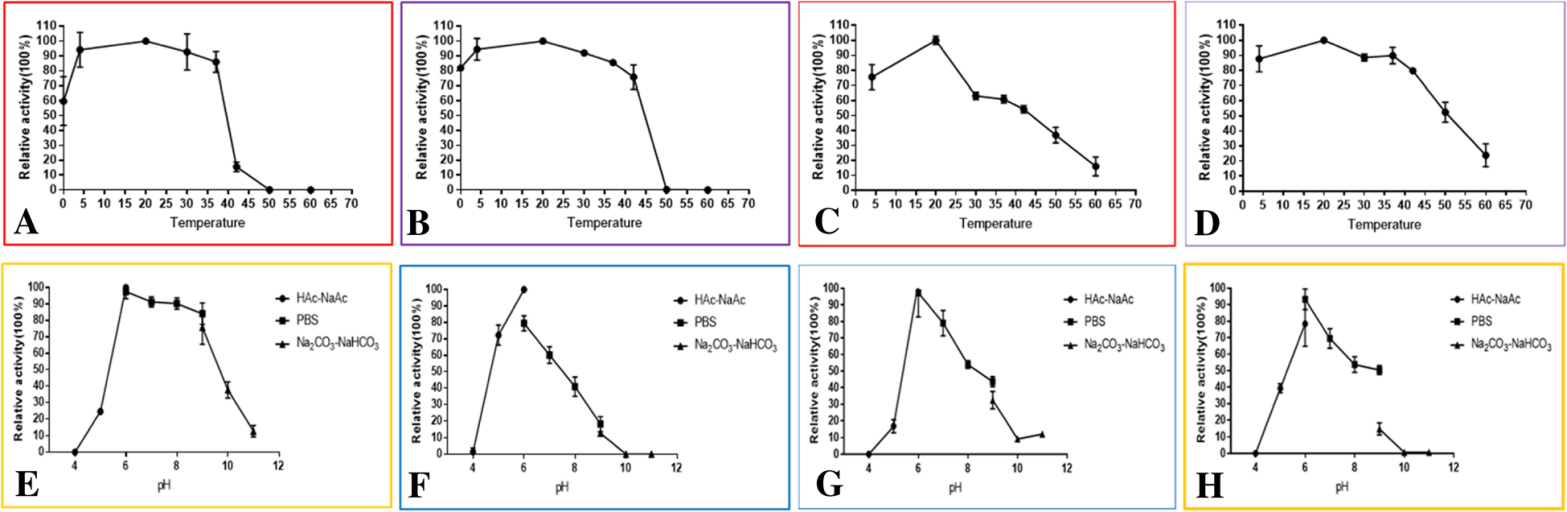
The effects of temperature (**a**-**d**) and pH (**e**-**h**) on the enzymatic activities. **a**, **c**, the effect of temperature on FLS (**a**) and F3H activities (**c**) of OcFLS1; **b**, **d**, the effect of temperature on FLS (**b**) and F3H activities (**d**) of OcFLS2; **e**, **g**, the effect of pH on FLS (**e**) and F3H activities (**g**) of OcFLS1; **f**, **h**, the effect of pH on FLS (**f**) and F3H activities (**h**) of OcFLS2. The data are presented as means ± standard deviation values of three biological replicates

The effect of temperature on FLS activity on these two enzymes was similar (Fig. 4). Both enzymes exhibited optimal activity at approximately 20 °C (Fig. 4). The two enzymes were cold-tolerant, both maintaining at least 60% activity at 0 °C. However, the two enzymes were completely inactivated at 50 °C (Fig. 4). The pH profiles towards FLS activity of the two enzymes were differentially displayed (Fig. 4). The two proteins displayed optimal FLS activity at pH 6.0. When the pH exceeded 6.0, the FLS activity of OcFLS1 decreased slightly (Fig. 4). When pH was 9.0, OcFLS1 retained approximately 80% activity (Fig. 4). As pH continued to rise, the OcFLS1 activity decreased significantly. When pH reached 11.0, the residual activity of OcFLS1 was approximately 10% (Fig. 4). Conversely, when pH was over 6.0, the FLS activity of OcFLS2 decreased remarkably (Fig. 4). When pH was 10.0, OcFLS2 completely lost its FLS activity (Fig. 4).

**Fig. 5:**
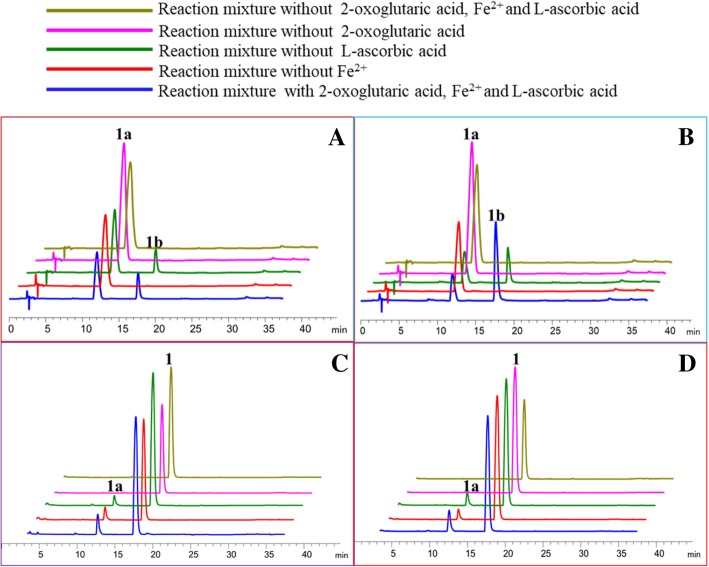
The cofactor-dependences of OcFLS1 (**a**, **c**) and OcFLS2 (**b**, **d**). **a**, **c**, the influence of cofactors on FLS (**a**) or F3H activity (**c**) of OcFLS1; **b**, **d**, the influence of cofactors on FLS (**b**) or F3H activity (**d**) of OcFLS2

Cofactor-dependencies of FLS activity of the two enzymes were investigated using dihydrokaempferol (**1a**) as the substrate (Fig. 5). The omission of Fe^2+^ and (or) 2-oxoglutaric acid resulted in the complete loss of FLS activity of the two enzymes, suggesting Fe^2+^ and (or) 2-oxoglutaric acid were required for the activity of both FLS (Fig. 5-a and -b). The omission of L-ascorbic acid did not result in a significant reduction in the activity of OcFLS1 and OcFLS2 (Fig. 5-a and -b). The cofactor-dependence of the two OcFLS proteins was somewhat different from that of flavonol synthase from poplars (PFLS). When reaction mixtures contained no 2-oxoglutaric acid and (or) L-ascorbic acid, PFLS activity was completely abolished. Conversely, PFLS retained 35% activity in the absence of Fe^2+^, indicating that Fe^2+^ was not absolutely required for PFLS activity [26].

**Fig. 6:**
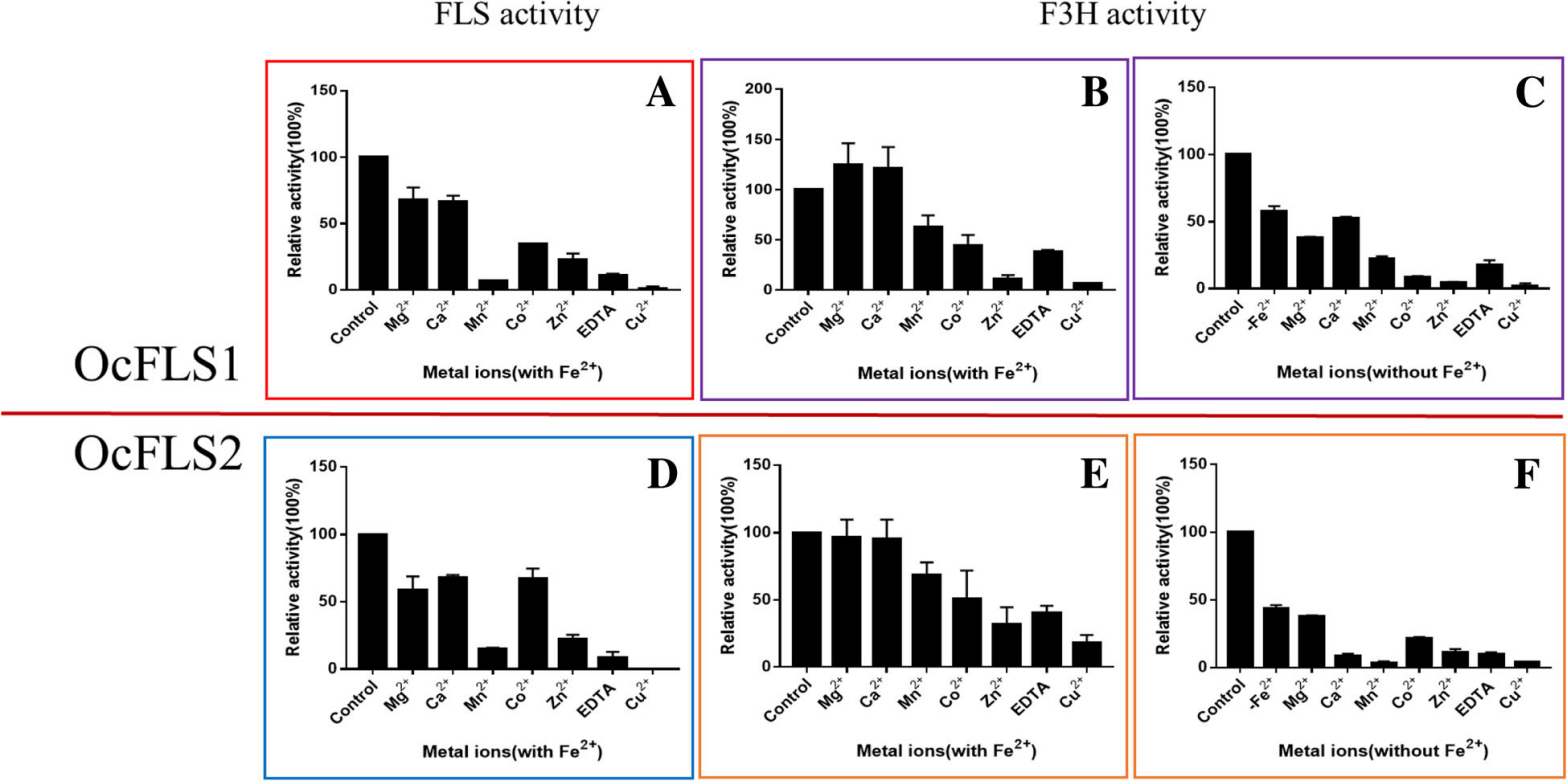
The effect of metal ions and chelating agents on activities of OcFLS1 and OcFLS2. **a**, **d**, The effect of metal ions and chelating agents on FLS activity of OcFLS1 (**a**) and OcFLS2 (**d**) in the presence of Fe^2+^. **b**, **e**, The effect of metal ions and chelating agents on F3H activity of OcFLS1 (**b**) and OcFLS2 (**e**) in the presence of Fe^2+^. The enzymatic activities in buffers containing only Fe^2+^ were set to 100%. **c**, **f**, The effect of metal ions and chelating agents on F3H activity of OcFLS1 (**c**) and OcFLS2 (**f**) in the absence of Fe^2+^. The enzymatic activities in buffers without any metal ions were set to 100%. The data are presented as means ± standard deviation values of three biological replicates

The effect of metal ions and chelating agents on FLS activity of the two enzymes was conducted in optimal pH and temperature. The inhibitory effect of these metal ions towards FLS activity of the two enzymes was observable (Fig. 6). Of these tested metal ions, Cu^2+^ displayed the most potent inhibitory effect on the two enzymes, both approaching 0 (Fig. 6).

Kinetic properties of FLS activity of OcFLS1 and OcFLS2 towards dihydrokaempferol (**1a**) were further examined at optimal pH and temperature. The apparent *K*_m_ and V_max_ for dihydrokaempferol (**1a**) calculated from Lineweaver-Burk plots are summarised in Table 1.

**Table 1:** Kinetic parameters of OcFLS1 and OcFLS2

	OcFLS1		OcFLS2	
	FLS activity	F3H activity	FLS activity	F3H activity
*K*_m_ (μM)	471.3 ± 179	37.57 ± 6.316	525.7 ± 198.4	110.5 ± 20.86
V_max_ (μM/h)	771.3 ± 172.7	123.1 ± 5.549	903.9 ± 206.8	349 ± 24.42

### Flavanone 3-hydroxylase activity of OcFLSs

**Fig. 7:**
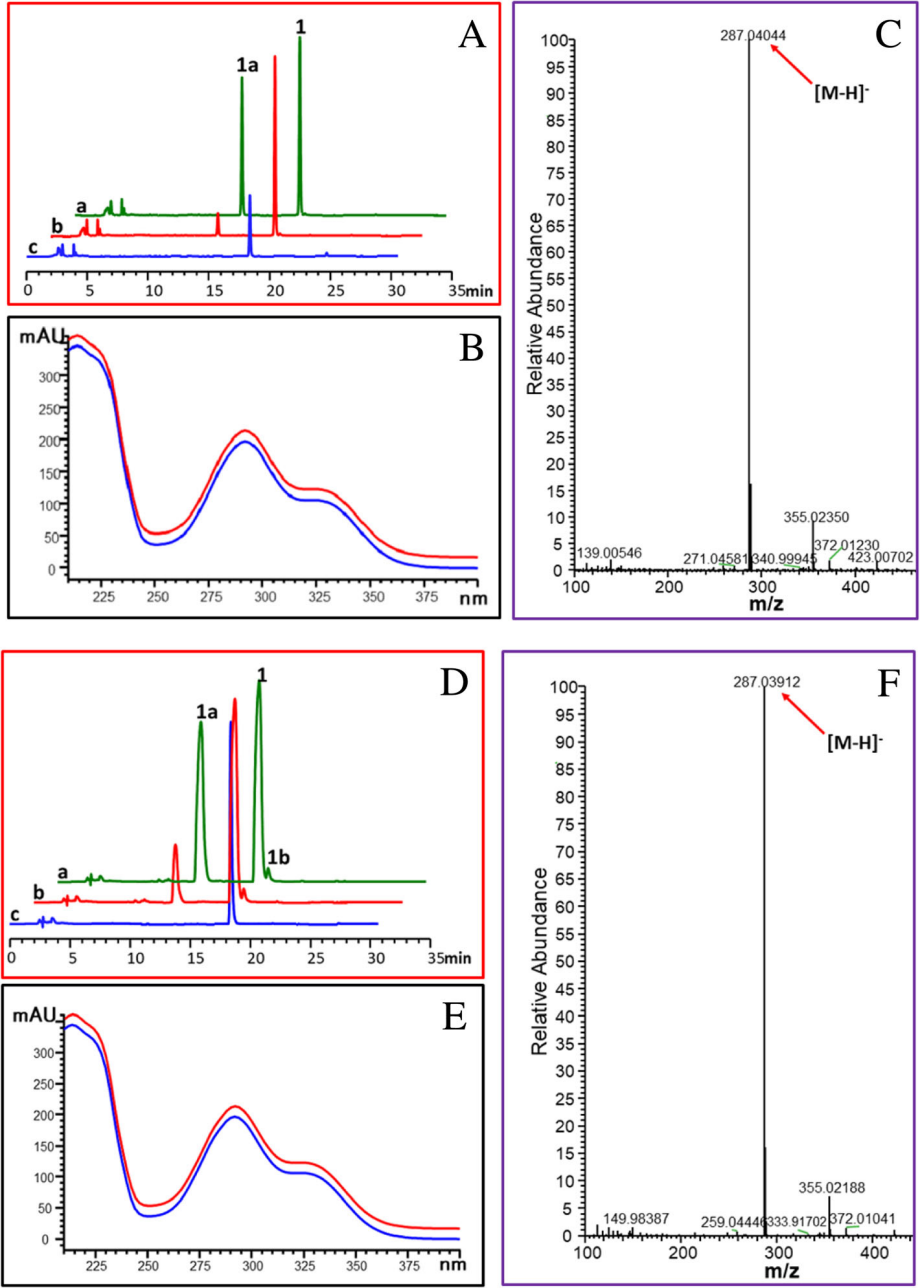
The conversion from (*S*)-naringenin (**1**) to dihydrokaempferol (**1a**) catalysed by OcFLS1 (**a**-**c**) and OcFLS2 (**d**-**f**) in the presence of Fe^2+^. **a**, **d**: HPLC chromatogram of the reaction product of (*S*)-naringenin (**1**) with OcFLS1 (**a**) and OcFLS2 (**d**). **a**, co-elution of the reaction product with authentic dihydrokaempferol (**1a**). **b**, the reaction product of (*S*)-naringenin (**1**) with the purified protein. **c**, the reaction product of (*S*)-naringenin (**1**) without the purified protein. **b**, **e**: UV spectrum of reaction product **1a** (blue line) and authentic dihydrokaempferol (red line). **c**, **f**: MS spectrum of reaction product **1a** produced by OcFLS1 (**c**) and OcFLS2 (**f**)

Flavonol synthase was demonstrated to be a bifunctional dioxygenase, either converting dihydroflavonols to flavonols or catalysing the 3-hydroxylation of flavanones to dihydroflavonols [18]. Hence, the flavanone 3-hydroxylase activity of OcFLS1 and OcFLS2 was also investigated. (*S*)-naringenin (**1**) was selected as a representative flavanone to react with recombinant OcFLS proteins. As shown in Fig. 7, after incubation of (*S*)-naringenin (**1**) with OcFLS1, a new peak was detected in the reaction mixture. The newly formed product displayed a similar UV spectrum as dihydrokaempferol (**1a**), suggesting it shared a similar skeleton structure with dihydrokaempferol (**1a**). Moreover, the newly formed compound showed a [M-H]^−^ peak at *m/z* 287.04044, indicating that it was a monohydroxylated product of (*S*)-naringenin (**1**). Further co-elution of this new metabolite with standard dihydrokaempferol (**1a**) confirmed the newly formed compound was dihydrokaempferol (**1a**) (Fig. 7). Likewise, OcFLS2 could catalyse (*S*)-naringenin (**1**) to form dihydrokaempferol (**1a**) (Fig. 7). Also, these two enzymes were demonstrated to catalyse 3β-hydroxylation towards (*S*)-pinocembrin (**2**), (*S*)-eriodictyol (**3**), and (*S*)-homoeriodictyol (**4**), thereby forming their respective dihydroflavonol products pinobanksin (**2a**) (Additional file 9: Figure S9), dihydroquercetin (**3a**) (Additional file 10: Figure S10), and 3′-*O*-methyltaxifolin (**4a**) (Additional file 11: Figure S11), respectively. These data indicated both OcFLS1 and OcFLS2 had F3H activity.

The effect of temperature upon F3H activity of these two enzymes was similar. As illustrated in Fig. 4, both OcFLSs displayed wide temperature tolerance and showed optimal activities at 20 °C. When the temperature dropped to 4 °C, the F3H activity of both enzymes was still high, approaching 90%. However, when the temperature was higher than 20 °C, the F3H activity of the two enzymes decreased dramatically. When the temperature reached 60 °C, the residual F3H activity of the two enzymes was less than 20%. Both of the enzymes showed optimal F3H activity at an acidic pH of 6.0 (Fig. 4), which is lower than values reported for F3H enzymes from *Petunia hybrida* [38], *Arabidopsis* [39], and *Artemisia annua* L [40]. As shown in Fig. 4, the F3H activities of OcFLS1 and OcFLS2 declined steeply when the pH exceeded 6.0.

The dependencies of cofactors 2-oxoglutaric acid, Fe^2+^, and L-ascorbic acid on F3H activity of OcFLSs were also investigated with purified enzymes (Fig. 5). The omission of 2-oxoglutaric acid resulted in almost complete loss of activity for both of the OcFLS enzymes (Figs. 5-c and -d). However, when L-ascorbic acid or Fe^2+^ was not present in the reaction mixture, the F3H activity of the two proteins did not disappear completely, but decreased slightly, suggesting L-ascorbic acid or Fe^2+^ was not absolutely necessary for the F3H activity of OcFLS1 and OcFLS2 (Fig. 5-c and -d). The L-ascorbic acid- and Fe^2+^-independent property of the two FLS enzymes was inconsistent with that of the single F3H enzymes reported previously, which were deemed L-ascorbic acid- and Fe^2+^-dependent oxygenases [38, 40, 41].

Considering that Fe^2+^ is not required for F3H activity, the influence of metal ions and chelating agents on F3H activity of the two enzymes was determined in the reaction systems with or without Fe^2+^ (Fig. 6). When the reaction mixtures contained no Fe^2+^, the additions of metal ions and chelating agent resulted in the reduction of F3H activities of OcFLS1 and OcFLS2. When Cu^2+^ was added into the reaction mixture, F3H activity of OcFLS1 and OcFLS2 was almost completely lost. Alternatively, in the buffer containing iron ions, the effects of metal ions on these two proteins were different. When Mn^2+^, Co^2+^, Zn^2+^, EDTA-2Na, or Cu^2+^ was added to the buffer containing Fe^2+^, the F3H activity of OcFLS1 and OcFLS2 was inhibited to varying degrees. Moreover, Mg^2+^ and Ca^2+^ had a slight inhibitory effect on F3H activity of OcFLS2. Conversely, these two metal ions displayed stimulating effects on F3H activity of OcFLS1 in the presence of Fe^2+^ (Fig. 6).

The *K*_m_ and V_max_ values of OcFLS1 and OcFLS2 towards (*S*)-naringenin (**1**) was calculated by Lineweaver-Burk plots (Table 1). The apparent Michaelis constants of F3H activity were lower than the corresponding values of FLS activity of OcFLS1 and OcFLS2, suggesting the affinity of the two flavonol synthases to (*S*)-naringenin (**1**) was stronger than to dihydrokaempferol (**1a**).

### Fe^2+^ is conducive to the bioconversion of flavanones to flavonols

**Fig. 8:**
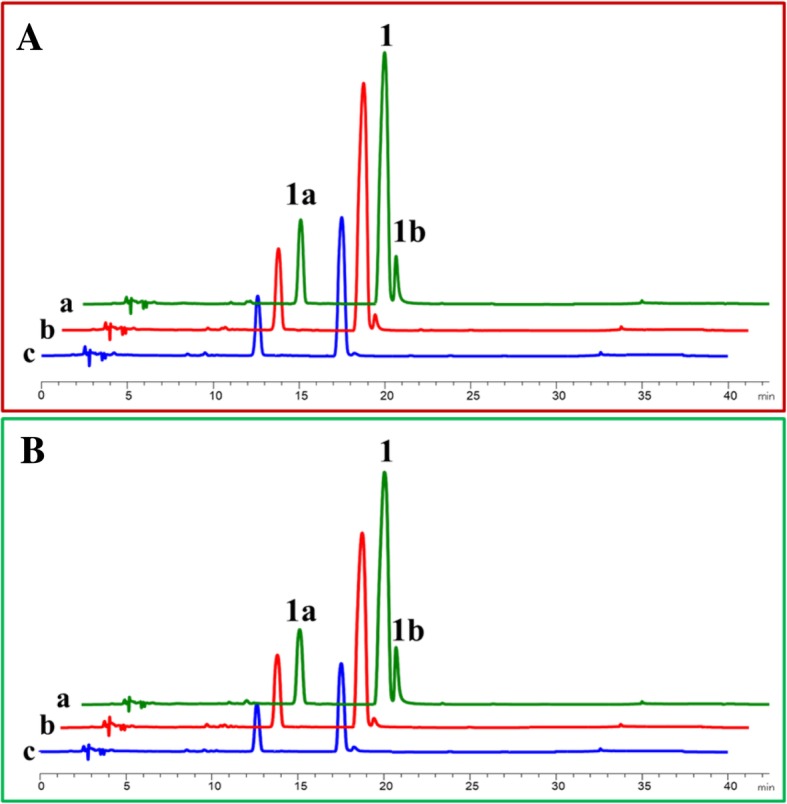
OcFLS1- (**a**) and OcFLS2-catalysed bioconversion (**b**) of (*S*)-naringenin (**1**) to form dihydrokaempferol (**1a**) and kaempferol (**1b**). **a**, the reaction was conducted for 4 h and was supplemented with ferrous sulphate at 2 h. **b**, the reaction was conducted for 4 h and was not supplemented with ferrous sulphate. **c**, the reaction was conducted for 2 h and was not supplemented with ferrous sulphate

OcFLS1 and OcFLS2 were demonstrated to be bifunctional enzymes, either catalysing dihydroflavonols to flavonols or performing the conversion of flavanones to dihydroflavonols. Thus, under the action of OcFLS1 or OcFLS2, flavanones could be converted to flavonols by two successive reactions (Fig. 1). However, only a small amount or no flavonols were detected in OcFLS1- or OcFLS2-catalysed transformations of flavanones (Fig. 7 and Additional file 9: Figure S9 Additional file 10: Figure S10 Additional file 11: Figure S11). As shown in Fig. 7, OcFLS1 catalysed (*S*)-naringenin (**1**) to form dihydrokaempferol (**1a**) but did not further catalyse dihydrokaempferol (**1a**) to generate kaempferol (**1b**) (Fig. 7a). In the reaction containing OcFLS2 and (*S*)-naringenin (**1**), although both **1a** and **1b** were detected, the yield of **1b** was relatively low (Fig. 7d). The catalytic effects of OcFLS1 and OcFLS2 on (*S*)-naringenin (**1**) and other flavanones showed that OcFLSs could not catalyse the formation of corresponding flavonols from flavanones as effectively as conceived. This inconsistency was likely related to a decrease in Fe^2+^ in the reaction mixture. When Fe^2+^ was added to the solution, it would react with oxygen in the air to form Fe^3+^, resulting in a decrease of Fe^2+^. Fe^2+^ is not absolutely required for the F3H activity of OcFLSs, and the reduction of Fe^2+^ therefore did not substantially affect F3H activity. Thus, a certain proportion of flavanones are converted to form dihydroflavonols (Fig. 7 and Additional file 9: Figure S9 Additional file 10: Figure S10 Additional file 11: Figure S11). Conversely, Fe^2+^ is absolutely required for the FLS activity of OcFLSs, and the decrease in Fe^2+^ would therefore lead to the reduction of the FLS activity of OcFLSs, in turn resulting in a low or undetectable amount of flavonols in the reaction mixture (Fig. 7 and Additional file 9: Figure S9 Additional file 10: Figure S10 Additional file 11: Figure S11). To test this speculation, OcFLS1- and OcFLS2-catalysed bioconversion of (*S*)-naringenin (**1**) were examined. The reaction mixtures were supplemented with 5 μl ferrous sulphate (20 mM) after 2 h of incubation at 20 °C and were then allowed to continue to incubate for an additional 2 h. The results showed that the yield of **1b** in the reaction mixtures supplemented with ferrous sulphate was significantly higher that of the control, indicating that Fe^2+^ played an important role in the continuous catalysis of OcFLSs (Fig. 8). Moreover, prolonging the reaction time from 2 h to 4 h could increase the yield of **1b** (Fig. 8).

### Expression profiles of OcFLS1 and OcFLS2 in various tissues

**Fig. 9:**
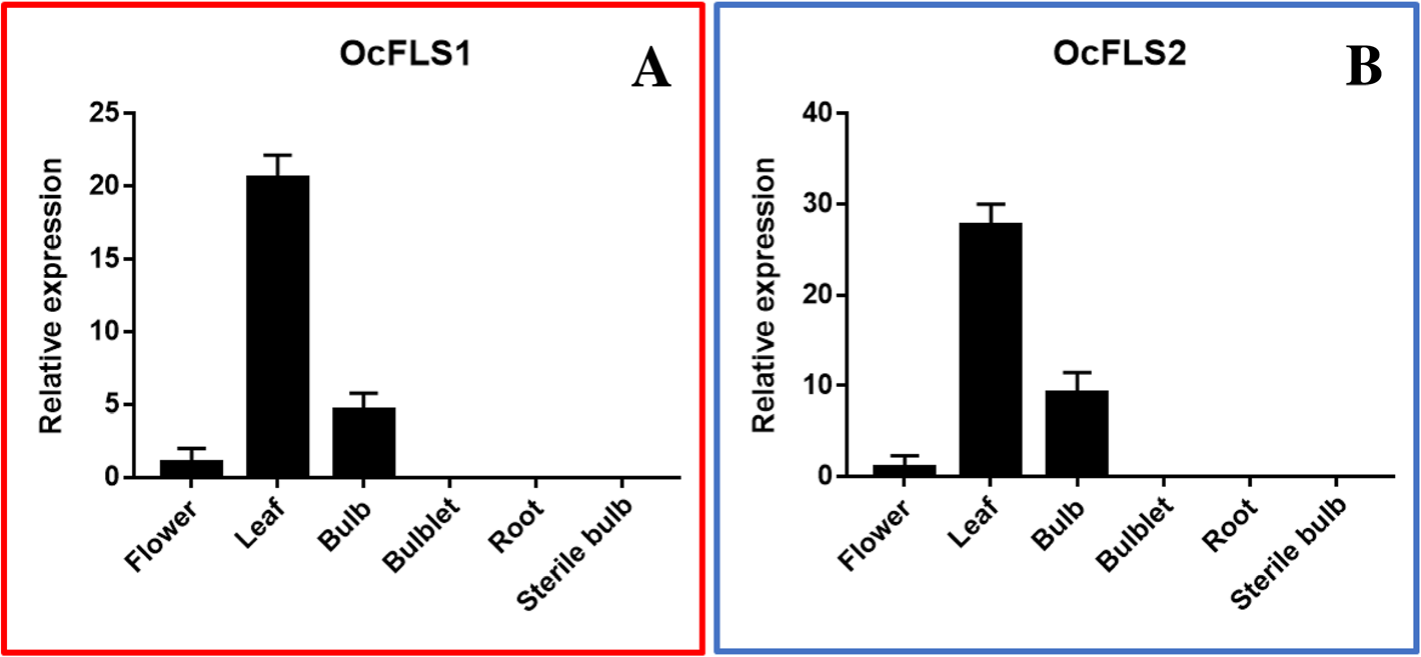
Relative transcript levels of OcFLS family members (**a**, OcFLS1; **b**, OcFLS2) in various tissues of *O. caudatum*, including flowers, leaves, bulbs, bulblets, roots, and sterile bulbs. The transcript level of each gene in flowers was defined as 1, and the relative expression in other tissues was calculated by means of 2^-∆∆CT^. Each value is the mean ± SD of three determinations

The expression profiles of OcFLS1 and OcFLS2 in *O. caudatum* were investigated by qRT-PCR analyses. As illustrated in Fig. 9, OcFLS1 and OcFLS2 were expressed in leaves, bulbs, and flowers. The highest expression levels of OcFLS1 and OcFLS2 were observable in leaves, followed by that in bulbs (Fig. 9). OcFLS1 and OcFLS2 showed the lowest expression level in flowers. Conversely, the two proteins were hardly expressed in roots, bulblets, and sterile bulbs (Fig. 9). Bulblets are young bulbs. The differential expression levels of OcFLS1 and OcFLS2 in bulblets and bulbs indicated that their expression was developmentally regulated. The sterile bulbs were bulbs cultivated in sterile and dark conditions. After successive subcultures, these sterile bulbs turned into white bulbs. The varied expression levels of OcFLS and OcFLS2 in bulbs and sterile bulbs revealed that their expression was regulated by environmental conditions. Cumulatively, the expression of these two OcFLS genes was regulated by developmental and environmental conditions.

## Discussion

*O. caudatum* is a medicinal plant [42–46]. Several active flavonols have been detected in this species [32]. However, the mining of enzymes related to flavonol biosynthesis is relatively poorly implemented. In this investigation, an FLS gene family containing two members were first isolated from *O. caudatum* based on transcriptome mining and then was functionally characterised by in vitro experiments. Data revealed the two flavonol synthases, OcFLS1 and OcFLS2, were not just capable of catalysing dihydroflavonols to form flavonols, but also could mediate the oxidation of flavanones to corresponding dihydroflavonols, an activity normally associated with an F3H enzyme. Flavonol synthase is the key controller of flavonol biosynthesis. Hence, the successful cloning and functional identification of OcFLS1 and OcFLS2 are conducive to pathway resolution of flavonol biosynthesis in *O. caudatum*.

In addition, detailed biochemical analyses of OcFLS1 and OcFLS2 are required to facilitate their application as genes used for the pathway reconstruction of flavonol biosynthesis in engineered strains. Flavonols are important sources of innovative drugs [1]. Preparation of flavonols is also faced with problems, such as cumbersome extraction and difficult synthesis. Therefore, our team has been devoted to the synthesis of flavonols by synthetic biological technology; that is, reconstructing biosynthetic pathways of flavonol biosynthesis in microorganisms such as *E. coli* and *Saccharomyces cerevisiae*, by introducing genes related to flavonol biosynthesis into these strains. These pathway enzymes need to be described in detail before they can be used as genetic elements for pathway reconstruction. Although bioinformatic tools can predict gene function quickly, this prediction is not particularly accurate. Owens et al. identified five candidate genes showing high similarity to flavonol synthase. However, only one FLS-like gene was demonstrated to encode a catalytically competent protein [47]. These data indicated that detailed biochemical study was required for the exact activity of flavonol synthase. Therefore, we performed a functional identification for OcFLS1 and OcFLS2. Obviously, these data have deepened our understanding of flavonol synthase and laid a solid foundation for the application of these two flavonol synthase genes in synthetic biology.

Flavonol synthase is a bifunctional dioxygenase, displaying both F3H-like and FLS activities [18, 48]. This bifunction makes flavonol synthase more widely used in synthetic biology. In the construction of engineering cells, only one *FLS* gene is needed to achieve the conversion from flavanones to flavonols, without introducing both *F3H* and *FLS* genes. With fewer foreign gene introductions, the metabolic burden of microorganisms is lower, which is conducive to the growth of engineered strains. Thus, it is necessary to thoroughly investigate the two activities of FLS. However, since the first discovery of bifunctional flavonol synthase [18, 48], there have been few comprehensive investigations on the bifunctional activity of flavonol synthase. Herein, we conducted thorough research on F3H-like and FLS activities of OcFLS1 and OcFLS2 and some interesting results were discovered. First, F3H-like and FLS activities of OcFLS1 and OcFLS2 were observed to respond differentially to Fe^2+^. Previous reports suggested that flavonol synthase is a Fe^2+^-dependent dioxygenase [18, 48, 49]. In fact, flavonol synthase requires Fe^2+^ only when it performs the FLS activity, but not absolutely when it carries out the F3H-like activity. In addition, we revealed that sufficient Fe^2+^ was necessary for the continuous catalysis of flavonols from flavanones. This result suggests that it is necessary to maintain sufficient Fe^2+^ in engineering cells for the conversion of flavonols from flavanones when constructing engineering cell factories.

In addition, the effects of metal ions and EDTA-2Na on the enzymatic activities of OcFLS1 and OcFLS2 were investigated in this study (Fig. 6). As shown in Fig. 6-c and -f, when other metal ions were added into the mixture without Fe^2+^, the F3H activity of OcFLS1 and OcFLS2 decreased. This may be caused by the complexation of these metal ions (1 mM) with flavonoids (0.5 mM) in the reaction mixture, which changes the structure of flavonoids and prevents the substrate from entering the reaction centre of the enzyme. When EDTA-2Na was added into the reaction mixture, the pH value of the reaction mixture decreased, thereby leading to a decline of F3H activity (Fig. 6-c and -f). If Fe^2+^ was added to the reaction mixture containing these metal ions or EDTA-2Na, ferrous iron could bind to enzymes as a common cofactor for activity improvement of the OcFLSs. Therefore, the enzymatic activity in the reaction solution harbouring Fe^2+^ is more than that in the reaction mixture without Fe^2+^, although the overall activity was still inhibited (Fig. 6-b and -e). In addition, because EDTA-2Na can chelate Fe^2+^, the introduction of EDTA-2Na into the reaction solution with Fe^2+^ can cause the decrease of F3H activity. Likewise, the addition of other metal ions and EDTA-2Na to the reaction solution containing Fe^2+^ also lead to a decrease in FLS activity of both enzymes (Fig. 6-a and -d).

## Conclusions

OcFLS1 and OcFLS2 were isolated from *O. caudatum* based on transcriptome mining and demonstrated to be bifunctional flavonol synthases, either converting dihydroflavonols to flavonols or catalysing 3-hydroxylation of flavanones to dihydroflavonols. Unlike the single F3H enzymes reported previously, which were deemed L-ascorbic acid- and Fe^2+^-dependent dioxygenases, OcFLS1 and OcFLS2 were determined to be L-ascorbic acid- and Fe^2+^-independent. In addition, sufficient Fe^2+^ was determined to be necessary for the continuous catalysis of flavonols from flavanones. The qRT-PCR analysis revealed their expression was regulated by developmental and environmental conditions.

## Methods

### Chemicals

The substrates listed in Additional file 8: Figure S8 are purchased from BioBioPha Co. Ltd. (Kunming, China) and Push Bio-Technology (Chengdu, China). Four flavanone substrates **1**–**4** were (2*S*)-enantiomers. The other chemicals were either reagent or analytic grade when available.

### Transcriptome-wide mining of unigenes coding for FLS

The transcriptome sequencing of *O. caudatum* was performed in our laboratory [46]. The resultant unigenes were aligned by Blast X to protein databases, including nr, Swiss-Prot, KEGG, and COG, thereby retrieving unigene encoding proteins showing the highest sequence similarity with flavonol synthase.

### Isolation of full-length FLS cDNAs and bioinformatic analyses of sequences

Whole plants of *O. caudatum* were cultivated in pots in our laboratory. The extraction and purification of total RNA of *O. caudatum* and subsequent cDNA synthesis were performed routinely [43]. The resulting cDNA was applied as the template to isolate sequences encoding FLS using unigene-specific primers (Additional file 12: Table S1) [45]. The amplified open reading frame (ORF) sequences of *OcFLS* genes were inserted into pEASY-Blunt (TransGen Co. Ltd., Beijing, China) to create recombinant constructs for sequencing (Additional file 12: Table S2). The obtained *OcFLS* sequences were analysed using online bioinformatic tools as previously described [50].

### Heterologous expression and protein purification

The full-length ORFs of *OcFLS* genes were amplified from their corresponding pEASY-Blunt derived plasmids and then inserted into *Eco*R I and *Hind* III sites of pET-28a (+) (Novagen, Madison, USA) using the ClonExpress II kit according to the manufacturer’s instructions (Vazyme, Nanjing, China), respectively. The resultant pET28a-OcFLS2 was transformed into *E. coli Trans*etta (DE3) (TransGen Co. Ltd., Beijing, China) for the recombinant expression. The other plasmid pET28a-OcFLS1 was co-transformed into *E. coli* BL21(DE3) strain with a chaperone plasmid pGro7 (Takara Biotechnology Co., Ltd., Dalian, China) to improve soluble expression as previously reported [42, 51]. These recombinant cells were cultivated until the optical density at 600 nm (OD_600_) reached 0.6 and was induced at 20 °C overnight by the addition of isopropyl-D-thiogalactopyranoside (IPTG) to reach a final concentration of 0.3 mM. The soluble expressions of *OcFLS* genes in *E. coli* were verified by sodium dodecyl sulphate polyacrylamide gel electrophoresis (SDS-PAGE). Next, the bacterially produced OcFLS proteins were purified and quantified as previously described [52, 53].

### Activity assays for OcFLSs

The enzymatic reactions were performed in PBS buffer (20 mM, pH 6.0) with a total volume of 200 μl containing α-ketoglutaric acid (1 mM), ferrous sulphate (0.5 mM), ascorbic acid (1 mM), 1 μl purified protein, and a substrate (0.5 mM). Incubations were conducted at 20 °C for 2 h and then stopped by the addition of methanol (100 μl) and acetic acid (10 μl). The mixture was mixed well and the solvent layer was separated by centrifugation at 12000 rpm for 10 min. The resulting solvent was filtrated through a 0.22 μm membrane. A 50 μl aliquot of solvent was analysed by high performance liquid chromatography (HPLC) and liquid chromatography-high resolution mass spectrometry (LC-HRMS).

### HPLC and LC-MS analyses

Reversed-phase HPLC was conducted on an Agilent 1200 HPLC system (Agilent Technologies Co. Ltd., CA, USA) with a SilGreen-C18 column (5 μm particle size, 4.6 × 250 mm; Beijing Greenherbs Science and Technology Development CO., Ltd., Beijing, China). HPLC conditions are summarised in Additional file 12: Table S3.

LC-MS analyses were conducted using an LC-MS system consisting of an Orbitrap mass spectrometer (Exactive, ThermoFisher Scientific, Inc., Bremen, Germany) coupled with a U-HPLC system (Accela, ThermoFisher Scientific, Inc., Bremen, Germany). The Orbitrap-MS was fitted with an integrated electrospray ionisation (ESI) source operating in negative ion mode. All data were acquired with full MS scan mode ranging from *m/z* 100 to 1000.

### Determination of enzymatic properties

The optimal temperature for enzymatic activity was investigated in PBS buffer (pH 6.0) at 4 °C, 20 °C, 30 °C, 37 °C, 42 °C, 50 °C, and 60 °C for 2 h. The dependence of enzymatic activity on pH was examined at 20 °C in HAc-NaAc buffer (pH 4.0–6.0), PBS buffer (pH 6.0–9.0) and Na_2_CO_3_-NaHCO_3_ (pH 9.0–11.0) for 2 h. The effects of cofactors (α-ketoglutaric acid, ferrous sulphate, and ascorbic acid) and metal ions (EDTA-2Na, MnCl_2_, MgCl_2_, CaCl_2_, CoCl_2_, ZnCl_2_, and CuCl_2_) were determined in PBS buffer (pH 6.0) at 20 °C for 2 h. The final concentration of each metal ion is 1 mM. Measurements were made in triplicate.

Kinetic characteristics were tested in optimal pH and temperature using eight different concentrations of the substrate (20 mM, 10 mM, 5 mM, 2.5 mM, 1.25 mM, 0.625 mM, 0.312 mM, and 0.156 mM). The apparent kinetic parameters (*K*_m_ and V_max_) were determined by means of Lineweaver-Burk plots derived from the Michaelis-Menten equation.

### qRT-PCR analysis

Quantitative reverse transcription-polymerase chain reaction (qRT-PCR) was performed to analyse the expression profiles of OcFLS1 and OcFLS2 in diverse tissues including roots, bulbs, leaves, flowers, sterile bulbs, and bulblets [44, 50]. Glyceraldehyde-3-phosphate dehydrogenase (GAPDH2, GenBank accession number KM370884) was used as the internal reference gene. The gene-specific primers are listed in Additional file 12: Table S1. The detailed procedure was the same as previously described [42, 44, 50]. The relative quantification of OcFLS expression was calculated using the 2^-ΔΔCt^ method.

## Additional files

Additional file 1: **Figure S1.** Schematic representation of unigenes showing sequence identity with FLS genes. (DOC 143 kb)

Additional file 2: **Figure S2.** Nested-PCR amplification of OcFLS cDNAs. Lane 1, PCR product of OcFLS1 (A) or OcFLS2 (B); Lane M, DNA molecular markers indicated in bp on the left side. (DOC 296 kb)

Additional file 3: **Figure S3.** Amino acid sequence alignment of OcFLS1 and OcFLS2 with other FLS proteins. The putative ferrous iron (HXDX53H) and 2-oxoglutarate binding motifs (RXS) are marked with red and blue pentagram, respectively. (DOC 2483 kb)

Additional file 4: **Figure S4.** Phylogenetic tree analysis of OcFLS1 and OcFLS2 with other 2-ODD proteins with demonstrated functionality. The phylogenetic tree was constructed using the neighbor-joining method available in the MEGA5.1 program. The numbers indicate bootstrap values (10,000 replicates). (DOC 188 kb)

Additional file 5: **Figure S5.** The conversion from pinobanksin (**2a**) to galangin (**2b**) catalyzed by OcFLS1 (A-C) or OcFLS2 (D-F). A, D: HPLC chromatogram of reaction product of pinobanksin (**2a**) with OcFLS1 (A) or OcFLS2 (D). a, the reaction product of pinobanksin (**2a**) with purified protein. b, the reaction product of pinobanksin (**2a**) without purified protein. B, E: UV spectrum of reaction product **2b**. C, F: MS spectrum of reaction product **2b**. (DOC 118 kb)

Additional file 6: **Figure S6.** The conversion from dihydroquercetin (**3a**) to quercetin (**3b**) catalyzed by OcFLS1 (A-C) or OcFLS2 (D-F). A, D: HPLC chromatogram of reaction product of dihydroquercetin (**3a**) with OcFLS1 (A) or OcFLS2 (D). a, the reaction product of dihydroquercetin (**3a**) with purified protein. b, the reaction product of dihydroquercetin (**3a**) without purified protein. B, E: UV spectrum of reaction product **3b**. C, F: MS spectrum of reaction product **3b**. (DOC 133 kb)

Additional file 7: **Figure S7.** The conversion from taxifolin 3′-methyl ether (**4a**) to isorhamnetin (**4b**) catalyzed by OcFLS1 (A-C) or OcFLS2 (D-F). A, D: HPLC chromatogram of reaction product of taxifolin 3′-methyl ether (**4a**) with OcFLS1 (A) or OcFLS2 (D). a, the reaction product of taxifolin 3′-methyl ether (**4a**) with purified protein. b, the reaction product of taxifolin 3′-methyl ether (**4a**) without purified protein.B, E: UV spectrum of reaction product **4b**. C, F: MS spectrum of reaction product **4b**. (DOC 124 kb)

Additional file 8: **Figure S8.** The compounds used in this study. (DOC 94 kb)

Additional file 9: **Figure S9.** The conversion from (*S*)-pinocembrin (**2**) to pinobanksin (**2a**) catalyzed by OcFLS1 (A-C) or OcFLS2 (D-F). A, D: HPLC chromatogram of reaction product of (*S*)-pinocembrin (**2**) with OcFLS1 (A) or OcFLS2 (D). a, the reaction product of (*S*)-pinocembrin (**2**) with purified protein. b, the reaction product of (*S*)-pinocembrin (**2**) without purified protein. B, E: UV spectrum of reaction product **2a**. C, F: MS spectrum of reaction product **2a**. (DOC 142 kb)

Additional file 10: **Figure S10.** The conversion from (*S*)-eriodictyol (**3**) to dihydroquercetin (**3a**) catalyzed by OcFLS1 (A-C) or OcFLS2 (D-F). A, D: HPLC chromatogram of reaction product of (*S*)-eriodictyol (**3**) with OcFLS1 (A) or OcFLS2 (D). a, the reaction product of (*S*)-eriodictyol (**3**) with purified protein. b, the reaction product of (*S*)-eriodictyol (**3**) without purified protein. B, E: UV spectrum of reaction product **3a**. C, F: MS spectrum of reaction product **3a**. (DOC 152 kb)

Additional file 11: **Figure S11.** The conversion from (*S*)-homoeriodictyol (**4**) to taxifolin 3′-methyl ether (**4a**) catalyzed by OcFLS1 (A-C) or OcFLS2 (D-F). A, D: HPLC chromatogram of reaction product of (*S*)-homoeriodictyol (**4**) with OcFLS1 (A) or OcFLS2 (D). a, the reaction product of (*S*)-homoeriodictyol (**4**) with purified protein.b, the reaction product of (*S*)-homoeriodictyol (**4**) without purified protein. B, E: UV spectrum of reaction product **4a**. C, F: MS spectrum of reaction product **4a**. (DOC 149 kb)

Additional file 12: **Table S1.** Primers used in this research. **Table S2** Plasmids and strains used in this investigation. **Table S3** HPLC conditions used in this study. (DOC 59 kb)

## Abbreviations

2-ODD: 2-oxoglutarate-dependent dioxygenases
aa: amino acids
ANS: Anthocyanidin synthase
DFR: Dihydroflavonol 4-reductase
F3H: Flavanone 3-hydroxylase
FLS: Flavonol synthase
FSI: Flavone synthase I
GAPDH: Glyceraldehyde-3-phosphate dehydrogenase
HPLC: High performance liquid chromatography
IPTG: Isopropyl β-D-thiogalactoside
LC-HRMS: Liquid chromatography-high resolution mass spectrometry
ORF: Open reading frame
pI: isoelectric point
qRT-PCR: Quantitative reverse transcription-polymerase chain reaction
SDS-PAGE: Sodium dodecyl sulfate-polyacrylamide gel electrophoresis
UTR: Untranslated region

## References

[1] Perez-Vizcaino F, Duarte J (2010). Flavonols and cardiovascular disease. Mol Asp Med.

[2] Hopia A, Heinonen M (1999). Antioxidant activity of flavonol aglycones and their glycosides in methyl linoleate. J Am Oil Chem Soc.

[3] Hichri F, Salah NB, Omri A, Hossan ASM, Jannet HB (2018). New antioxidant C-glycosyl flavone and flavonol derivatives from the Tunisian *Achille acretica* L. S Afr J Bot.

[4] Corsino J, Silva DHS, Zanoni MVB, Bolzani VDS, França SC, Pereira AMS, Furlan M (2003). Antioxidant flavan-3-ols and flavonol glycosides from *Maytenus aquifolium*. Phytother Res.

[5] Jiménez M, García-Carmona F (1999). Myricetin, an antioxidant flavonol, is a substrate of polyphenol oxidase. J Sci Food Agr.

[6] Marzouk MS, Moharram FA, Haggag EG, Ibrahim MT, Badary OA (2006). Antioxidant flavonol glycosides from *Schinus molle*. Phytother Res.

[7] Britton RG, Horner-Glister E, Pomenya OA, Smith EE, Denton R, Jenkins PR (2012). Synthesis and biological evaluation of novel flavonols as potential anti-prostate cancer agents. Eur J Med Chem.

[8] Dias TA, Duarte CL, Lima CF, Proença MF, Pereira-Wilson C (2013). Superior anticancer activity of halogenated chalcones and flavonols over the natural flavonol quercetin. Eur J Med Chem.

[9] Li S, Dong P, Wang J, Zhang J, Gu J, Wu X (2010). Icariin, a natural flavonol glycoside, induces apoptosis in human hepatoma SMMC-7721 cells via a ROS/JNK-dependent mitochondrial pathway. Cancer Lett.

[10] Granica S, Czerwińska ME, Żyżyńska-Granica B, Kiss AK (2013). Antioxidant and anti-inflammatory flavonol glucuronides from *Polygonum aviculare* L. Fitoterapia..

[11] Ortega YH, Foubert K, Berghe WV, Chirumamilla CS, Pieters L, Mosquera DMG, Apers S (2017). Flavonol glycosides from the leaves of *Boldoa purpurascens* and their anti-inflammatory properties. Phytochem Lett.

[12] Tahiri O, Atmani-Kilani D, Sanchez-Fidalgo S, Aparicio-Soto M, Alarcón-de-la-Lastra C, Barrajón-Catalán E (2017). The flavonol-enriched *Cistus albidus* chloroform extract possesses in vivo anti-inflammatory and anti-nociceptive activity. J Ethnopharmacol.

[13] Li Y, Ding Y (2012). Minireview: therapeutic potential of myricetin in diabetes mellitus. Food Sci Hum Well.

[14] Sendrayaperumal V, Pillai SI, Subramanian S (2014). Design, synthesis and characterization of zinc–morin, a metal flavonol complex and evaluation of its antidiabetic potential in HFD–STZ induced type 2 diabetes in rats. Chem Biol Interact.

[15] Şöhretoğlu D, Sari S, Barut B, Özel A (2018). Discovery of potent α-glucosidase inhibitor flavonols: insights into mechanism of action through inhibition kinetics and docking simulations. Bioorg Chem.

[16] Preuß A, Stracke R, Weisshaar B, Hillebrecht A, Matern U, Martens S (2009). *Arabidopsis thaliana* expresses a second functional flavonol synthase. FEBS Lett.

[17] Nakatsuka T, Abe Y, Kakizaki Y, Yamamura S, Nishihara M (2007). Production of red-flowered plants by genetic engineering of multiple flavonoid biosynthetic genes. Plant Cell Rep.

[18] Lukačin R, Wellmann F, Britsch L, Martens S, Matern U (2003). Flavonol synthase from *Citrus unshiu* is a bifunctional dioxygenase. Phytochemistry..

[19] Holton TA, Brugliera F, Tanaka Y (1993). Cloning and expression of flavonol synthase from *Petunia hybrida*. Plant J.

[20] Britsch L, Heller W, Grisebach H (1981). Conversion of flavanone to flavone, dihydroflavonol and flavonol with an enzyme system from cell cultures of parsley. Z Naturforsch C.

[21] Liu W, Xiao Z, Fan C, Jiang N, Meng X, Xiang X (2018). Cloning and characterization of a flavonol synthase gene from *Litchi chinensis* and its variation among litchi cultivars with different fruit maturation periods. Front Plant Sci.

[22] Akita Y, Kitamura S, Mikami R, Ishizaka H (2018). Identification of functional *flavonol synthase* genes from fragrant wild cyclamen (*Cyclamen purpurascens*). J Plant Biochem Biot.

[23] Kallscheuer N, Vogt M, Bott M, Marienhagen J (2017). Functional expression of plant-derived *O*-methyltransferase, flavanone 3-hydroxylase, and flavonol synthase in *Corynebacterium glutamicum* for production of pterostilbene, kaempferol, and quercetin. J Biotechnol.

[24] Park SR, Paik JH, Ahn MS, Park JW, Yoon YJ (2010). Biosynthesis of plant-specific flavones and flavonols in *Streptomyces venezuelae*. J Microbiol Biotechnol.

[25] Yang SM, Han SH, Kim BG, Ahn JH (2014). Production of kaempferol 3-*O*-rhamnoside from glucose using engineered *Escherichia coli*. J Ind Microbiol Biot.

[26] Kim BG, Joe EJ, Ahn JH (2010). Molecular characterization of flavonol synthase from poplar and its application to the synthesis of 3-*O*-methylkaempferol. Biotechnol Lett.

[27] Miyahisa I, Funa N, Ohnishi Y, Martens S, Moriguchi T, Horinouchi S (2006). Combinatorial biosynthesis of flavones and flavonols in *Escherichia coli*. Appl Microbiol Biot.

[28] Leonard E, Yan Y, Koffas MAG (2006). Functional expression of a P450 flavonoid hydroxylase for the biosynthesis of plant-specific hydroxylated flavonols in *Escherichia coli*. Metab Eng.

[29] Rodriguez A, Strucko T, Stahlhut SG, Kristensen M, Svenssen DK, Forster J (2017). Metabolic engineering of yeast for fermentative production of flavonoids. Bioresour Technol.

[30] Duan L, Ding W, Liu X, Cheng X, Cai J, Hua E, Jiang H (2017). Biosynthesis and engineering of kaempferol in *Saccharomyces cerevisiae*. Microb Cell Factories.

[31] Trantas E, Panopoulos N, Ververidis F (2009). Metabolic engineering of the complete pathway leading to heterologous biosynthesis of various flavonoids and stilbenoids in *Saccharomyces cerevisiae*. Metab Eng.

[32] Tang YP, Yu B, Hu J, Wu T, Hui YZ (2001). The chemical constituents from the bulbs of *Ornithogalum caudatum*. J Chin Pharmaceut Sci.

[33] Toh HC, Wang SY, Chang ST, Chu FH (2013). Molecular cloning and characterization of flavonol synthase in *Acacia confusa*. Tree Genet Genomes.

[34] Li C, Bai Y, Li S, Chen H, Han X, Zhao H (2012). Cloning, characterization, and activity analysis of a flavonol synthase gene *FtFLS1* and its association with flavonoid content in tartary buckwheat. J Agr Food Chem.

[35] Sun ZX, Hou SY, Yang WD, Han YH (2012). Exogenous application of salicylic acid enhanced the rutin accumulation and influenced the expression patterns of rutin biosynthesis related genes in *Fagopyrum tartaricum Gaertn* leaves. Plant Growth Regul.

[36] Gebhardt Y, Witte S, Forkmann G, Lukacin R, Matern U, Martens S (2005). Molecular evolution of flavonoid dioxygenases in the family Apiaceae. Phytochemistry..

[37] Martens S, Forkmann G, Britsch L, Wellmann F, Matern U, Lukačin R (2003). Divergent evolution of flavonoid 2-oxoglutarate-dependent dioxygenases in parsley. FEBS Lett.

[38] Britsch L, Grisebach H (1986). Purification and characterization of (2*S*)-flavanone 3-hydroxylase from *Petunia hybrida*. Eur J Biochem.

[39] Owens DK, Crosby KC, Runac J, Howard BA, Winkel BSJ (2008). Biochemical and genetic characterization of *Arabidopsis* flavanone 3β-hydroxylase. Plant Physiol Bioch..

[40] Xiong S, Tian N, Long J, Chen Y, Qin Y, Feng J (2016). Molecular cloning and characterization of a flavanone 3-hydroxylase gene from *Artemisia annua* L. Plant Physiol Bioch.

[41] Kim JH, Lee YJ, Kim BG, Lim Y, Ahn J (2008). Flavanone 3β-hydroxylases from rice: key enzymes for favonol and anthocyanin biosynthesis. Mol Cells.

[42] Yin S, Sun YJ, Liu M, Li LN, Kong JQ (2016). cDNA isolation and functional characterization of UDP-d-glucuronic acid 4-epimerase family from *Ornithogalum caudatum*. Molecules..

[43] Yin S, Liu M, Kong JQ (2016). Functional analyses of OcRhS1 and OcUER1 involved in UDP-L-rhamnose biosynthesis in *Ornithogalum caudatum*. Plant Physiol Bioch..

[44] Yin S, Kong JQ (2016). Transcriptome-guided discovery and functional characterization of two UDP-sugar 4-epimerase families involved in the biosynthesis of anti-tumor polysaccharides in *Ornithogalum caudatum*. RSC Adv.

[45] Yin S, Kong JQ (2016). Transcriptome-guided gene isolation and functional characterization of UDP-xylose synthase and UDP-d-apiose/UDP-d-xylose synthase families from *Ornithogalum caudatum* Ait. Plant Cell Rep.

[46] Guo L, Chen X, Li LN, Tang W, Pan YT, Kong JQ (2016). Transcriptome-enabled discovery and functional characterization of enzymes related to (2*S*)-pinocembrin biosynthesis from *Ornithogalum caudatum* and their application for metabolic engineering. Microb Cell Factories.

[47] Owens DK, Alerding AB, Crosby KC, Bandara AB, Westwood JH, Winkel BSJ (2008). Functional analysis of a predicted flavonol synthase gene family in Arabidopsis. Plant Physiol.

[48] Prescott AG, Stamford NPJ, Wheeler G, Firmin JL (2002). In vitro properties of a recombinant flavonol synthase from *Arabidopsis thaliana*. Phytochemistry..

[49] Martens Stefan, Preuß Anja, Matern Ulrich (2010). Multifunctional flavonoid dioxygenases: Flavonol and anthocyanin biosynthesis in Arabidopsis thaliana L. Phytochemistry.

[50] Li Li-Na, Kong Jian-Qiang (2016). Transcriptome-wide identification of sucrose synthase genes in Ornithogalum caudatum. RSC Advances.

[51] Liu Ming, Kong Jian-Qiang (2018). The enzymatic biosynthesis of acylated steroidal glycosides and their cytotoxic activity. Acta Pharmaceutica Sinica B.

[52] Yuan Shuai, Liu Ming, Yang Yan, He Jiu-Ming, Wang Ya-Nan, Kong Jian-Qiang (2018). Transcriptome-Wide Identification of an Aurone Glycosyltransferase with Glycosidase Activity from Ornithogalum saundersiae. Genes.

[53] Liu Ming, Li Li-Na, Pan Yi-Ting, Kong Jian-Qiang (2017). cDNA isolation and functional characterization of squalene synthase gene from Ornithogalum caudatum. Protein Expression and Purification.

