# Supplementary figures and images for: Characterization of two flavonol synthases with iron-independent flavanone 3-hydroxylase activity from Ornithogalum caudatum Jacq
Source: BMC Plant Biol. 2019 May 14;19:195. doi: 10.1186/s12870-019-1787-x (PMC6515686; doi:10.1186/s12870-019-1787-x)

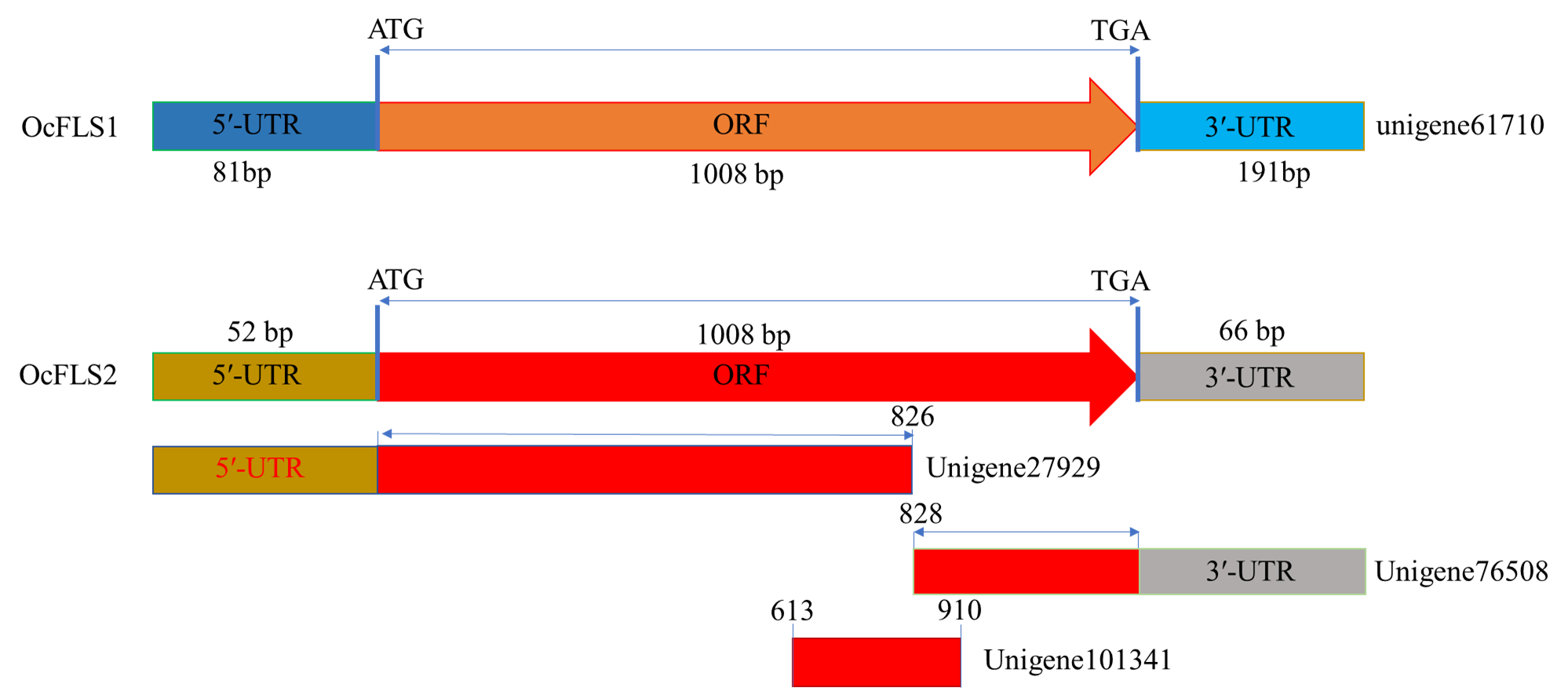


Figure S1

Supplement: Supplementary file 1 — Figure S1. Schematic representation of unigenes showing sequence identity with FLS genes. (DOC 143 kb) [file 12870_2019_1787_MOESM1_ESM.doc]

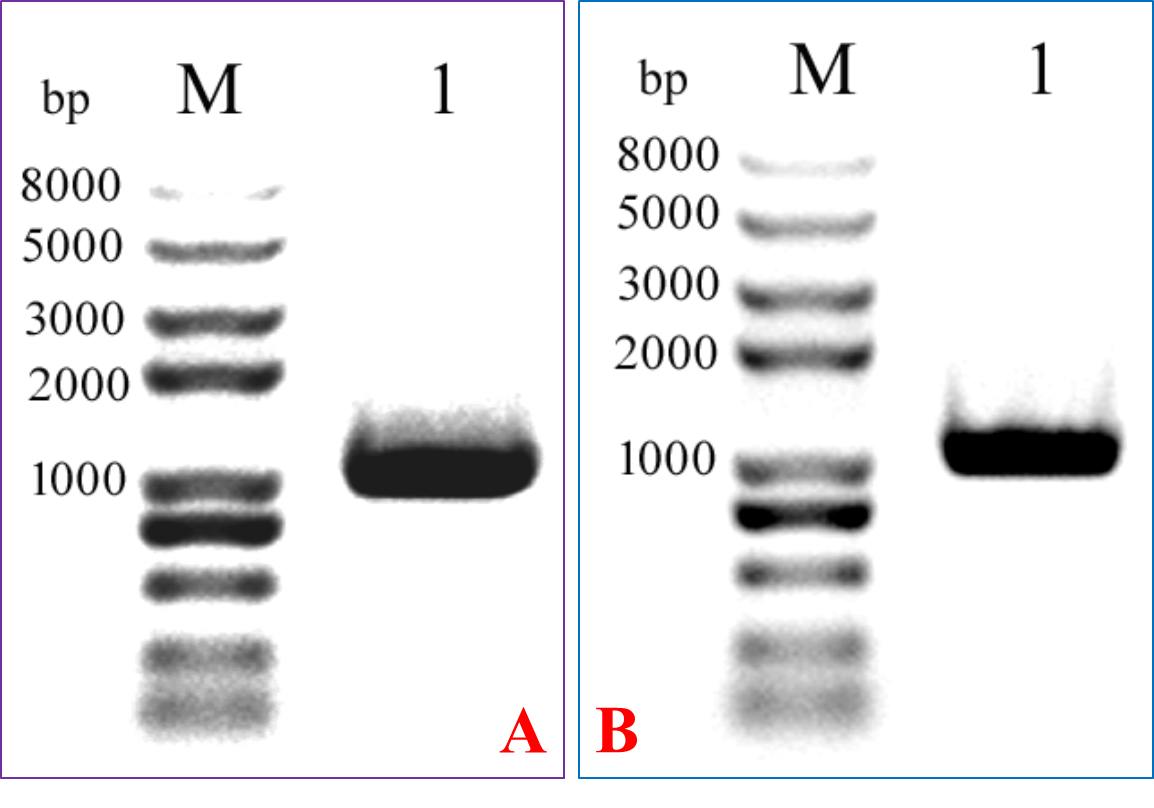


Figure S2

Supplement: Supplementary file 2 — Figure S2. Nested-PCR amplification of OcFLS cDNAs. Lane 1, PCR product of OcFLS1 (A) or OcFLS2 (B); Lane M, DNA molecular markers indicated in bp on the left side. (DOC 296 kb) [file 12870_2019_1787_MOESM2_ESM.doc]

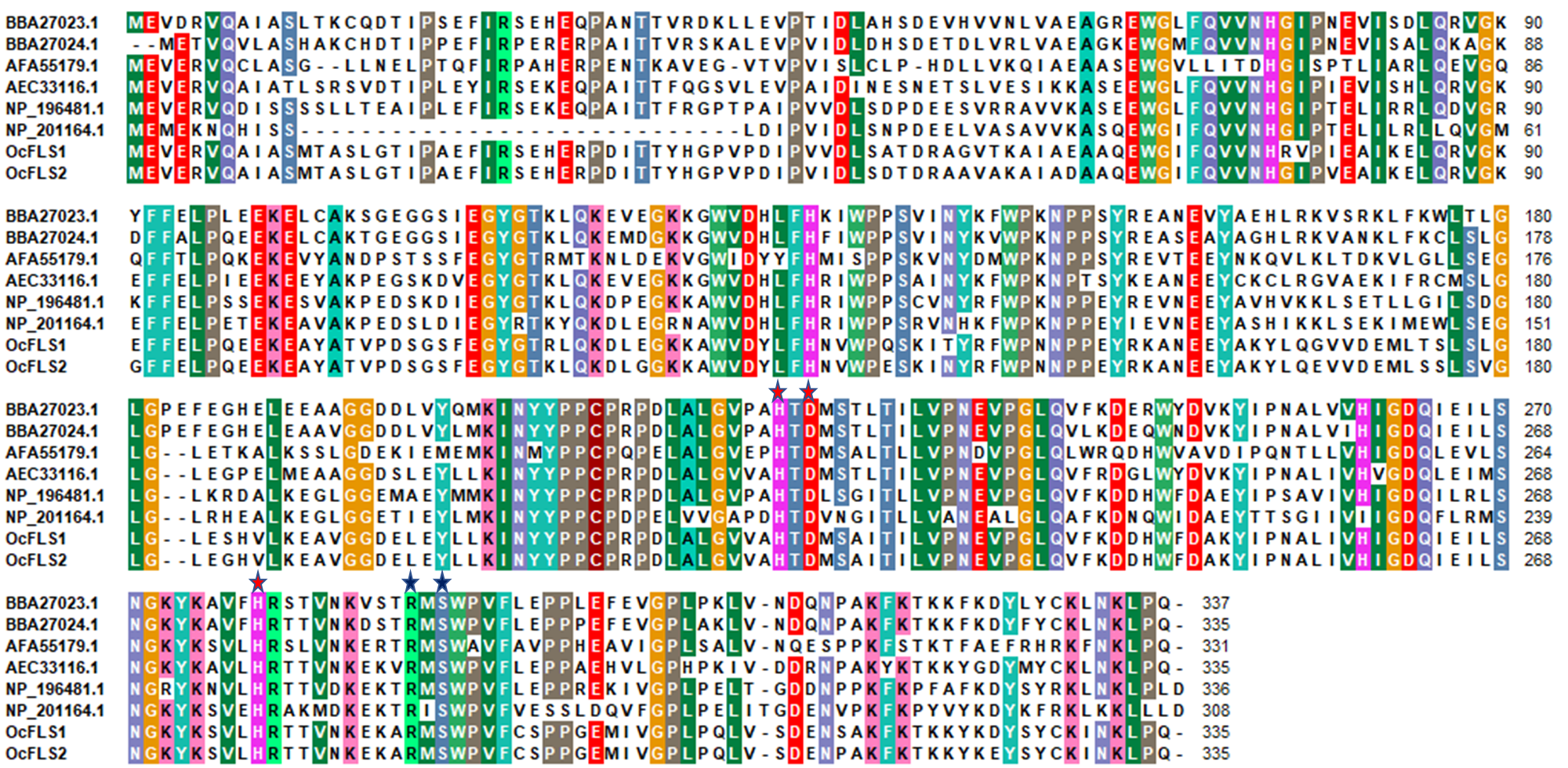


Figure S3

Supplement: Supplementary file 3 — Figure S3. Amino acid sequence alignment of OcFLS1 and OcFLS2 with other FLS proteins. The putative ferrous iron (HXDX53H) and 2-oxoglutarate binding motifs (RXS) are marked with red and blue pentagram, respectively. (DOC 2483 kb) [file 12870_2019_1787_MOESM3_ESM.doc]

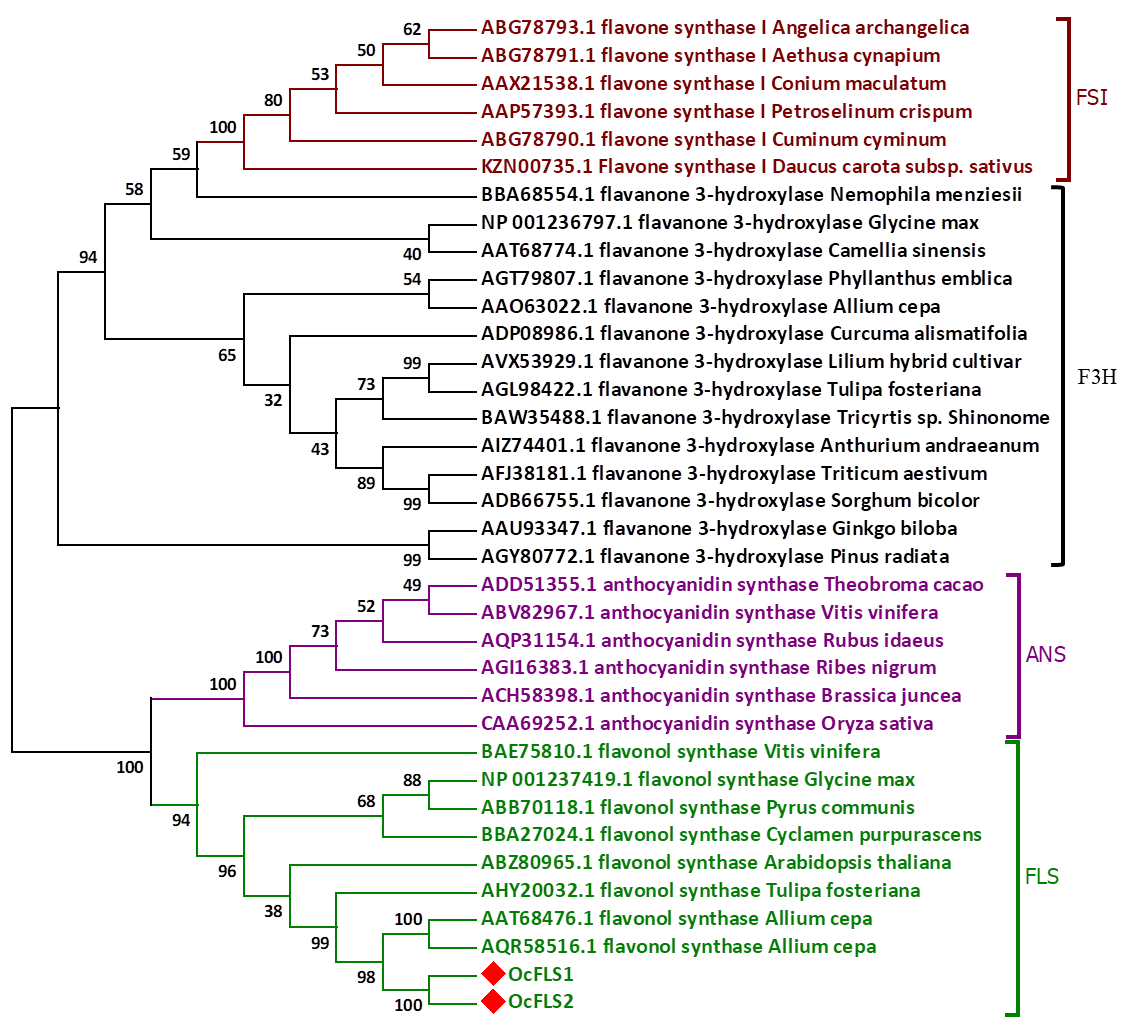


Figure S4

Supplement: Supplementary file 4 — Figure S4. Phylogenetic tree analysis of OcFLS1 and OcFLS2 with other 2-ODD proteins with demonstrated functionality. The phylogenetic tree was constructed using the neighbor-joining method available in the MEGA5.1 program. The numbers indicate bootstrap values (10,000 replicates). (DOC 188 kb) [file 12870_2019_1787_MOESM4_ESM.doc]

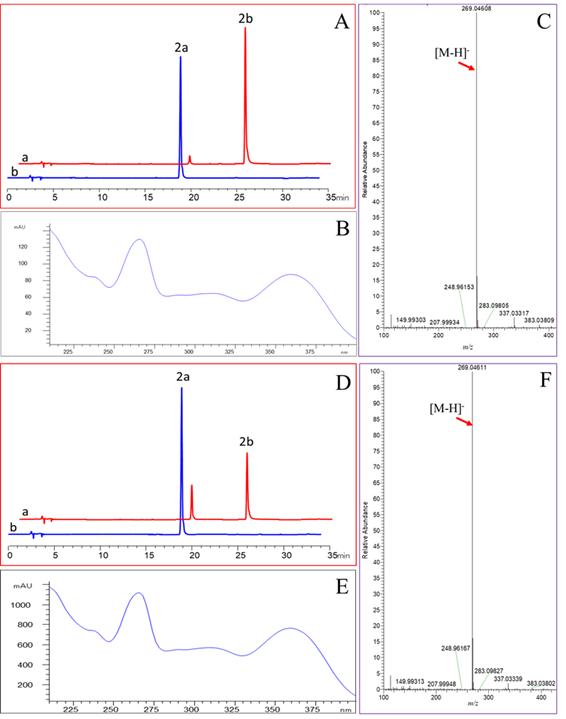


Figure S5

Supplement: Supplementary file 5 — Figure S5. The conversion from pinobanksin (2a) to galangin (2b) catalyzed by OcFLS1 (A-C) or OcFLS2 (D-F). A, D: HPLC chromatogram of reaction product of pinobanksin (2a) with OcFLS1 (A) or OcFLS2 (D). a, the reaction product of pinobanksin (2a) with purified protein. b, the reaction product of pinobanksin (2a) without purified protein. B, E: UV spectrum of reaction product 2b. C, F: MS spectrum of reaction product 2b. (DOC 118 kb) [file 12870_2019_1787_MOESM5_ESM.doc]

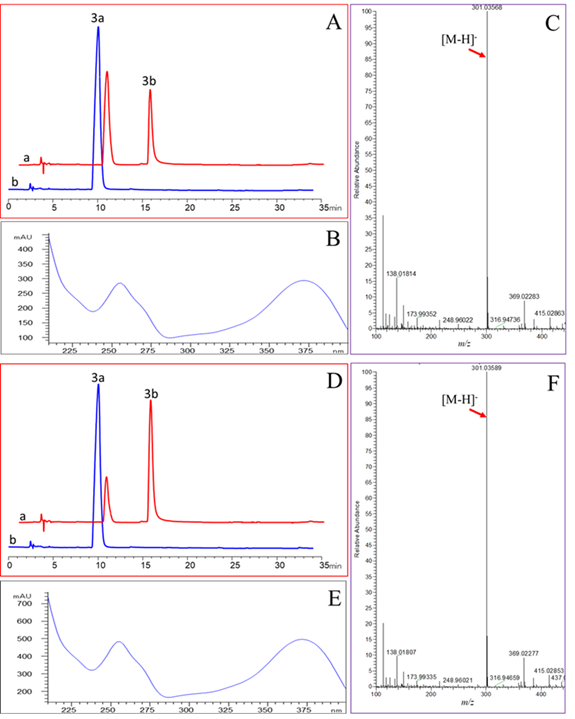


Figure S6

Supplement: Supplementary file 6 — Figure S6. The conversion from dihydroquercetin (3a) to quercetin (3b) catalyzed by OcFLS1 (A-C) or OcFLS2 (D-F). A, D: HPLC chromatogram of reaction product of dihydroquercetin (3a) with OcFLS1 (A) or OcFLS2 (D). a, the reaction product of dihydroquercetin (3a) with purified protein. b, the reaction product of dihydroquercetin (3a) without purified protein. B, E: UV spectrum of reaction product 3b. C, F: MS spectrum of reaction product 3b. (DOC 133 kb) [file 12870_2019_1787_MOESM6_ESM.doc]

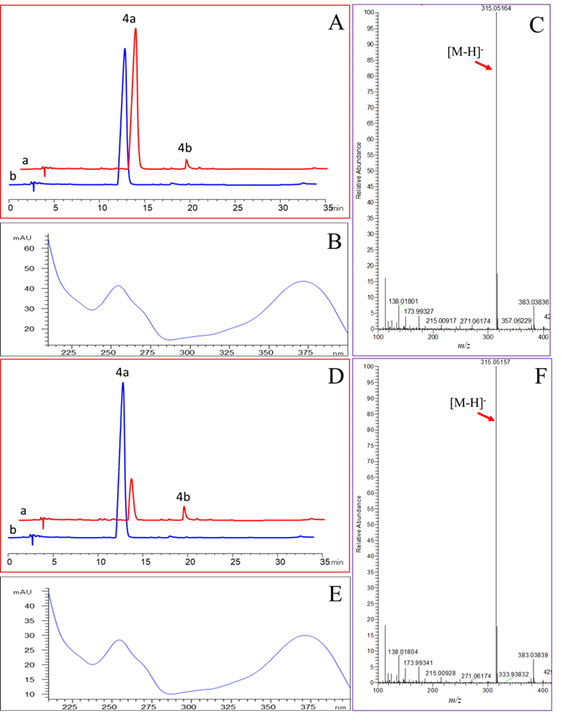


Figure S7

Supplement: Supplementary file 7 — Figure S7. The conversion from taxifolin 3′-methyl ether (4a) to isorhamnetin (4b) catalyzed by OcFLS1 (A-C) or OcFLS2 (D-F). A, D: HPLC chromatogram of reaction product of taxifolin 3′-methyl ether (4a) with OcFLS1 (A) or OcFLS2 (D). a, the reaction product of taxifolin 3′-methyl ether (4a) with purified protein. b, the reaction product of taxifolin 3′-methyl ether (4a) without purified protein.B, E: UV spectrum of reaction product 4b. C, F: MS spectrum of reaction product 4b. (DOC 124 kb) [file 12870_2019_1787_MOESM7_ESM.doc]

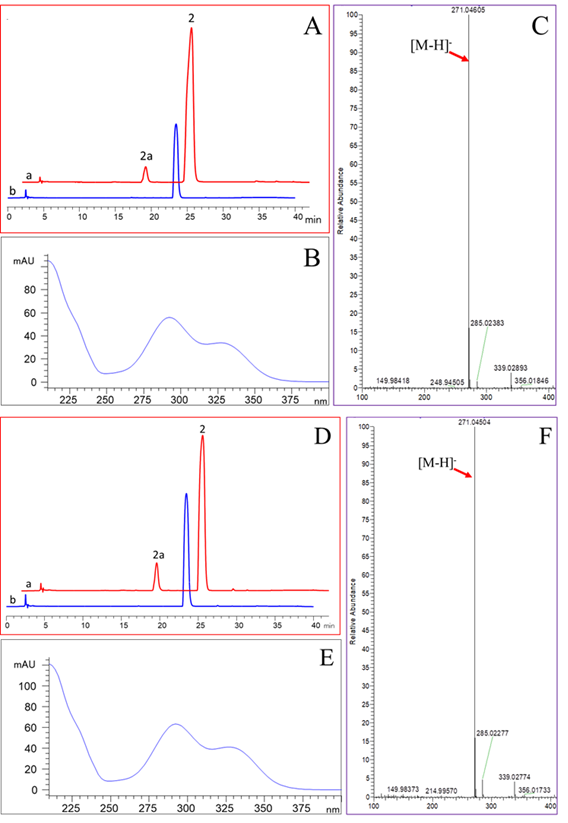


Figure S9

Supplement: Supplementary file 9 — Figure S9. The conversion from (S)-pinocembrin (2) to pinobanksin (2a) catalyzed by OcFLS1 (A-C) or OcFLS2 (D-F). A, D: HPLC chromatogram of reaction product of (S)-pinocembrin (2) with OcFLS1 (A) or OcFLS2 (D). a, the reaction product of (S)-pinocembrin (2) with purified protein. b, the reaction product of (S)-pinocembrin (2) without purified protein. B, E: UV spectrum of reaction product 2a. C, F: MS spectrum of reaction product 2a. (DOC 142 kb) [file 12870_2019_1787_MOESM9_ESM.doc]

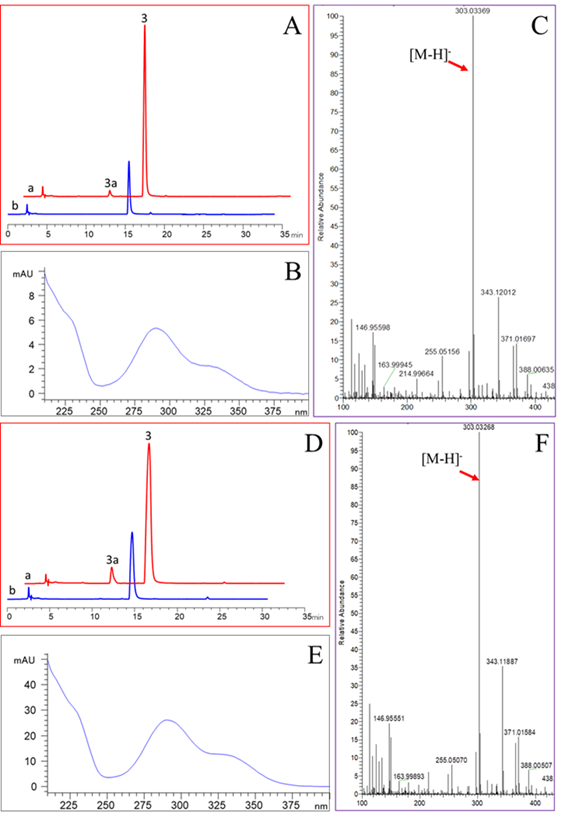


Figure S10

Supplement: Supplementary file 10 — Figure S10. The conversion from (S)-eriodictyol (3) to dihydroquercetin (3a) catalyzed by OcFLS1 (A-C) or OcFLS2 (D-F). A, D: HPLC chromatogram of reaction product of (S)-eriodictyol (3) with OcFLS1 (A) or OcFLS2 (D). a, the reaction product of (S)-eriodictyol (3) with purified protein. b, the reaction product of (S)-eriodictyol (3) without purified protein. B, E: UV spectrum of reaction product 3a. C, F: MS spectrum of reaction product 3a. (DOC 152 kb) [file 12870_2019_1787_MOESM10_ESM.doc]

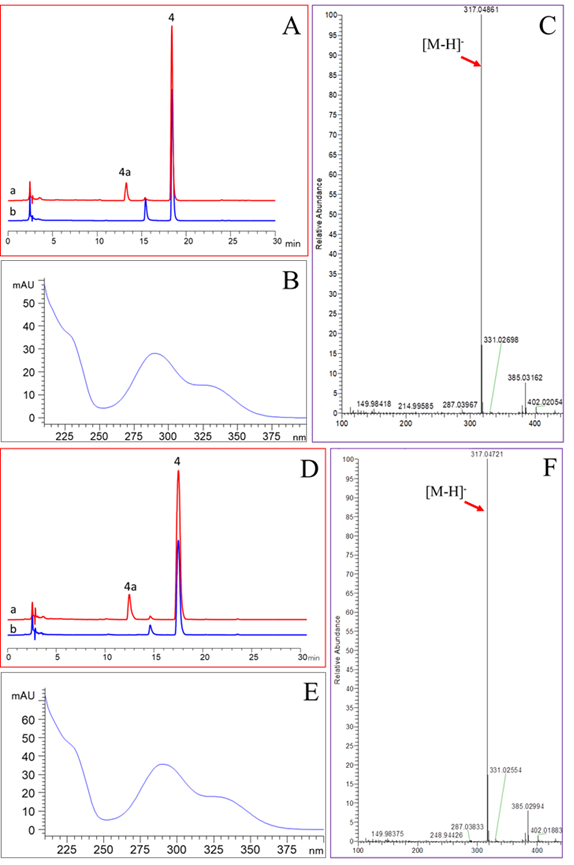


Figure S11

Supplement: Supplementary file 11 — Figure S11. The conversion from (S)-homoeriodictyol (4) to taxifolin 3′-methyl ether (4a) catalyzed by OcFLS1 (A-C) or OcFLS2 (D-F). A, D: HPLC chromatogram of reaction product of (S)-homoeriodictyol (4) with OcFLS1 (A) or OcFLS2 (D). a, the reaction product of (S)-homoeriodictyol (4) with purified protein.b, the reaction product of (S)-homoeriodictyol (4) without purified protein. B, E: UV spectrum of reaction product 4a. C, F: MS spectrum of reaction product 4a. (DOC 149 kb) [file 12870_2019_1787_MOESM11_ESM.doc]
