# Supplementary material for: Characterization of two flavonol synthases with iron-independent flavanone 3-hydroxylase activity from Ornithogalum caudatum Jacq
Source: BMC Plant Biol. 2019 May 14;19:195. doi: 10.1186/s12870-019-1787-x (PMC6515686; doi:10.1186/s12870-019-1787-x)
Supplement: Supplementary file 12 — Table S1. Primers used in this research. Table S2 Plasmids and strains used in this investigation. Table S3 HPLC conditions used in this study. (DOC 59 kb) [file 12870_2019_1787_MOESM12_ESM.doc]

**Table S1**

| Primer | Sequence (5' to 3') | Description |
| --- | --- | --- |
| F61710-1 | CTACCTGCAAATCAAAAACAAT | Forward primer used for OcFLS1 amplification in the first round |
| R61710-1 | CGATACATATATTTGCTAGCTC | Reverse primer used for OcFLS1 amplification in the first round |
| F61710-2 | ATGGAGGTGGAGAGAGTGC | Forward primer used for OcFLS1 amplification in the second round |
| R61710-2 | TCACTGTGGAAGCTTGTTG | Reverse primer used for OcFLS1 amplification in the second round |
| F271076-1 | ATCACAATGGAGGTAGAGAG | Forward primer used for OcFLS1 amplification in the first round |
| R271076-1 | GTATATTTGCTCTCACTGTGG | Reverse primer used for OcFLS1 amplification in the first round |
| F271076-2 | ATGGAGGTAGAGAGAGTGC | Forward primer used for OcFLS1 amplification in the second round |
| R271076-2 | TCACTGTGGAAGCTTATTGAT | Reverse primer used for OcFLS1 amplification in the second round |
| FqRTF3H1 | GGGTTGGGAAGGAGTTCTTCGAG | Forward primer used for qRT-PCR analysis of *OcFLS1* gene |
| RqRTF3H1 | CCGTGGGCAAGGCGGATAG | Reverse primer used for qRT-PCR analysis of *OcFLS1* gene |
| FqRTF3H2 | ACGATCCCAGCCGAGTTCATACG | Forward primer used for qRT-PCR analysis of *OcFLS2* gene |
| RqRTF3H2 | CCAAGCCTTCTTCCCTCCCAAAT | Reverse primer used for qRT-PCR analysis of *OcFLS2* gene |
| FGAPDH2 | ACTTGGTGTCCACCGACTTC | Forward primer used for qRT-PCR analysis of *GAPDH2* gene |
| RGAPDH2 | ATTCGTTGTCGTACCAAGCC | Reverse primer used for qRT-PCR analysis of *GAPDH2* gene |

**Table S2**

| Strains/plasmids | Description | Source/Reference |
| --- | --- | --- |
| Strain | | |
| *Trans*1-T1 | F^-^ φ80 (*lac*Z) ∆M15∆*lac*X74 *hsd*R (r_k_^-^,m_k_^+^) ∆*rec*A1398*end*A1*ton*A | TransGen, Beijing, China |
| *Trans*etta (DE3) | F^-^*omp*T*hsd*S_B_(r_B_^-^m_B_^-^)*gal dcm* (DE3)pRARE(argU, argW,ilex,glyT,leuW,proL)Cam^r^) | TransGen, Beijing, China |
| BL21(DE3) | F- ompT hsdS(rB-mB-) gal dcm(DE3) | TransGen, Beijing, China |
| BL21 (DE3)[pET28a-OcFLS1+pGro7] | BL21(DE3) derived strain harboring pET28aOcFLS1 and pGro7 | This study |
| BL21(DE3)[pET-28a(+)+pGro7] | BL21(DE3) derived strain harboring pET-28a(+) and pGro7 | This study |
| Plasmid | | |
| *pEASY*^TM^-Blunt | General cloning vector, T7 promoter, f1 ori, Amp^r^ and Kan^r^ | TransGen, Beijing, China |
| pET-28a (+) | General expression vector, T7 promoter, f1 ori, Kan^r^ | Novagen, Madison, USA |
| *pEASY-*OcFLS1 | *pEASY*^TM^-Blunt derived vector containing *OcFLS1* gene | This study |
| *pEASY-*OcFLS2 | *pEASY*^TM^-Blunt derived vector containing *OcFLS2* gene | This study |
| pET28a-OcFLS1 | pET-28a (+) derived vector containing *OcFLS1* gene | This study |
| pET28a-OcFLS2 | pET-28a (+) derived vector containing *OcFLS2* gene | This study |
| pET28a-MOcFLS2 | pET-28a (+) derived vector containing M*OcFLS2* gene | This study |

**Table S3**

| Method | Solvent A | Solvent B | Gradient | Substrate (detection wavelength) |
| --- | --- | --- | --- | --- |
| 1 | H_2_O | Acetonitrile | 15-70%B; 28min  70-100%B; 1min  100%B; 4min  100-15%; 7min | **1**, **1a**, **1b**, **2**, **2a**, **2b**, **3**, **3a**, **3b**, **4**, **4a**, **4b**, **5**, **9**, **10**, **11**, **12** (310 nm) |
| 2 | H_2_O | Acetonitrile | 56-66%B; 20min  66-90%B; 5min  90%B; 10min  90-56%; 1min  56%B; 6min | **8** (310nm) |
| 3 | H_2_O | Acetonitrile | 10-50%B;38min  50-100%B; 2min  100%B; 3min  100-10%B; 7min | **6**, **7** (280nm) |
| 4 | H_2_O | Acetonitrile | 11-21%B; 20min  21-90%B; 5min  90%B; 10min  90-11%B; 1min  11%B; 4min | **13**, **14**, **15** (280nm) |
